# A Bronze Age town in the Khaybar walled oasis: Debating early urbanization in Northwestern Arabia

**DOI:** 10.1371/journal.pone.0309963

**Published:** 2024-10-30

**Authors:** Guillaume Charloux, Shadi Shabo, Bruno Depreux, Sylvain Colin, Kévin Guadagnini, François Guermont, Sabine Dupuy, Mylène Bussy, Noisette Bec Drelon, Modwene Poulmarc’h, Diaa Albukaai, Saifi Alshilali, Rémy Crassard, Munirah AlMushawh

**Affiliations:** 1 French National Center for Scientific Research CNRS, UMR 8167, Orient et Méditerranée, Paris, France; 2 French National Center for Scientific Research CNRS, UMR 5133 Archéorient, Lyon, France; 3 Hadès Bureau d’investigation Archéologique, L’Union, France; 4 Independent Scholar, France; 5 Royal Commission for AlUla, Riyadh, Khaybar and AlUla, Saudi Arabia; Israel Antiquities Authority, ISRAEL

## Abstract

Recent exploration of the Khaybar oasis by the Khaybar Longue Durée Archaeological Project (AFALULA-RCU-CNRS) has led to the discovery of an exceptional Bronze Age fortified site called al-Natah. For the first time in Northwestern Arabia, the characteristics of a third/second-millennium-BCE settlement can be assessed over a large area. Preliminary archaeological survey and soundings have revealed a fortified 2.6-hectares town built around 2400–2000 BCE which lasted until at least 1500 BCE and possibly 1300 BCE−but with possible interruptions−, functionally subdivided into a residential area, a probable decision-making zone and a necropolis. The nucleated dwellings were constructed following a standard plan and were connected by small streets. By comparison with neighboring oasis centers, we suggest that Northwestern Arabia during the Bronze Age−largely dominated by pastoral nomadic groups and already integrated into long-distance trade networks−was dotted with interconnected monumental walled oases centered around small fortified towns. And by comparison with the contemporary situation in the Southern Levant, we also envisage that the archaeological record bears witness to a ‘low urbanization’ (or ‘slow urbanism’), indigenous to North Arabia, evidencing weak but increasing social complexity through the Early and Middle Bronze Ages.

## Introduction

**Fig 1:**
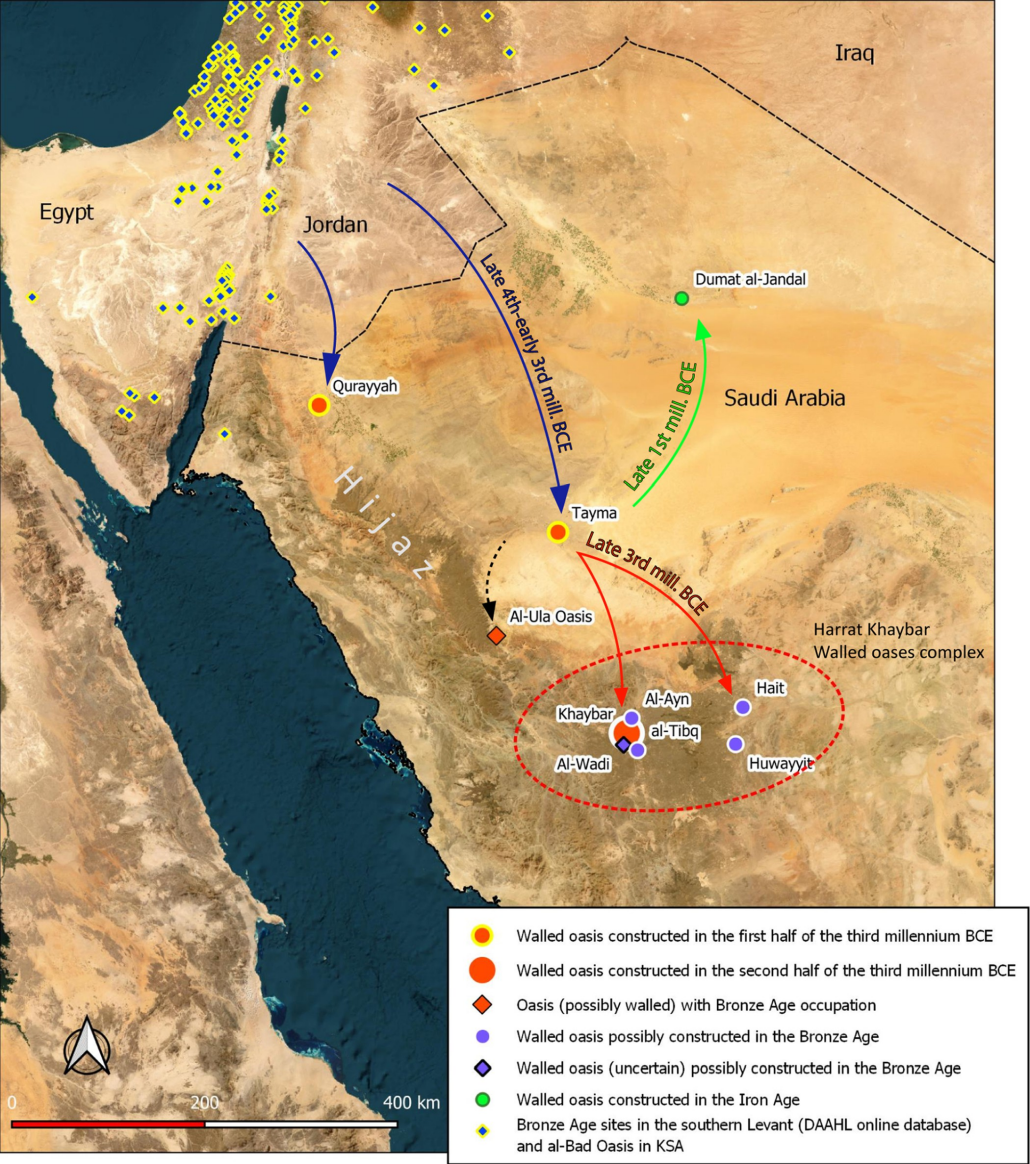
Bronze Age walled oases and main settlements in Northwestern Arabia, with the location of Khaybar Oasis and the Harrat Khaybar walled oases complex (dashed red oval). Source: Esri, DAAHL online Database, QGIS OSM.

The emergence of urbanization is a difficult research problem to tackle in Northwestern Arabia (the region between Mecca and Aqaba) (Fig 1) ([1–4]), in contrast to much better-known neighboring cultural spheres—Egypt and Mesopotamia, the Southern Levant, but also Southern and Eastern Arabia ([3, 5–8]: &4–5). In these cradles of civilization, the definitions and modes of urban development have given rise to intense debate for more than half a century. Inappropriately described as an ‘urban revolution’ in its day ([9]), urbanization in the Middle East at the turn of the Chalcolithic and Early Bronze Age was characterized by slow socio-economic change and a wide variety of urban trajectories depending on the different environmental and social niches ([10]). The urbanization of the Middle East is marked by strong regionalism and urban polymorphism, which sometimes involve divergences in ‘urban’ criteria used in other geographical areas, including size, density, socio-political organization, planning and function. The main argument adopted by scholars remains the identification of a high degree of social, political and economic stratification, indicating that a political entity was in control of the means of production and social and spatial organization ([11]: 225). However, for some researchers, the identification of urbanization is a kind of necessary outcome, as it signifies the attainment of urban state status by a society, which is considered to be characteristic of increased social complexity. The criteria adopted, often ‘revolutionary’, seem sometimes poorly suited to subsistence ways of life in marginal regions (e.g., [12]).

The fact that access to Saudi archaeological terrain was difficult until around twenty years ago (e.g., [13, 14]) amply accounts for the marginalization of Northwestern Arabia in reflections on the development of urbanization in the Middle East in the fourth to second millennia BCE (qualified here as the Bronze Age period), and more generally on the process of increased socio-economic complexity in this vast and long-unexplored territory. The lack of in-depth investigations of oases−today greatly impacted by rapid urban development (e.g., [15])−and the rare sedentary ancient settlements within them, have influenced the study and characterization of urbanism in the desert and the identification of its rhythms and modes of evolution. Prior to discoveries made by the Saudi-German mission in the Tayma oasis some twenty years ago ([13, 16]), the Northwestern Arabian Bronze Age had been little explored, glimpsed solely through the study of the funerary or megalithic structures dotting the deserts, and rock art representations dated with a significant margin of uncertainty (e.g., [17–21]). Those studies led scholars to believe that the region during the Bronze Age (fourth to late second mill. BCE) was an ‘Arabian Bronze Age Megalithic complex’ ([19]: 116, fig 6), a vast desert populated by groups of nomadic pastoralists who buried their dead in cairns. Cities were thought to appear after this long phase of nomadism, at the end of the Late Bronze Age, alongside the creation of local political entities and the emergence of caravan trade ([1, 22, 23]: 199). It was thus considered that the urbanization of Arabia emerged at the end of the second millennium, driven either by Egyptian colonizers of the New Kingdom ([1, 24]), or through difficult to characterize indigenous development ([2, 25]).

Over the last twenty years or so, the discovery of Bronze Age archaeological records in a large number of regional oases (Hegra, Tayma, Qurayyah, AlUla, Hait, Dumat al-Jandal, al-Bad) has considerably transformed our knowledge of contexts from the fourth to the second millennium BCE ([13, 26–33]). It is now fitting to investigate the emergence of urbanization specific to the oases of Northwestern Arabia. Scholars have indeed referred several times to the ‘urban’ character of oases at that time ([34]: 380; [16, 33]: 16; [4, 35]), notably evidenced by a complex of walled oases−defined as oases entirely enclosed by outer walls, comprising not only inhabited areas but the whole oasis territory, including rural zones ([33]). However, it is still difficult to assess the degree of organization and political integration of sedentary populations at those sites (particularly in the Early and Middle Bronze Age), as so little contextual archaeological data are available for these periods and no residential areas have yet been unearthed on a large scale in the region. Several alternative theoretical models developed in the Southern Levantine region (‘townism’, ‘slow urbanism’ [36], ‘heterarchy’ [4, 37, 38]: 109, ‘rural complexity’ [39]) could indeed better define the socio-political character of Northwestern Arabian walled oases at that time.

**Fig 2:**
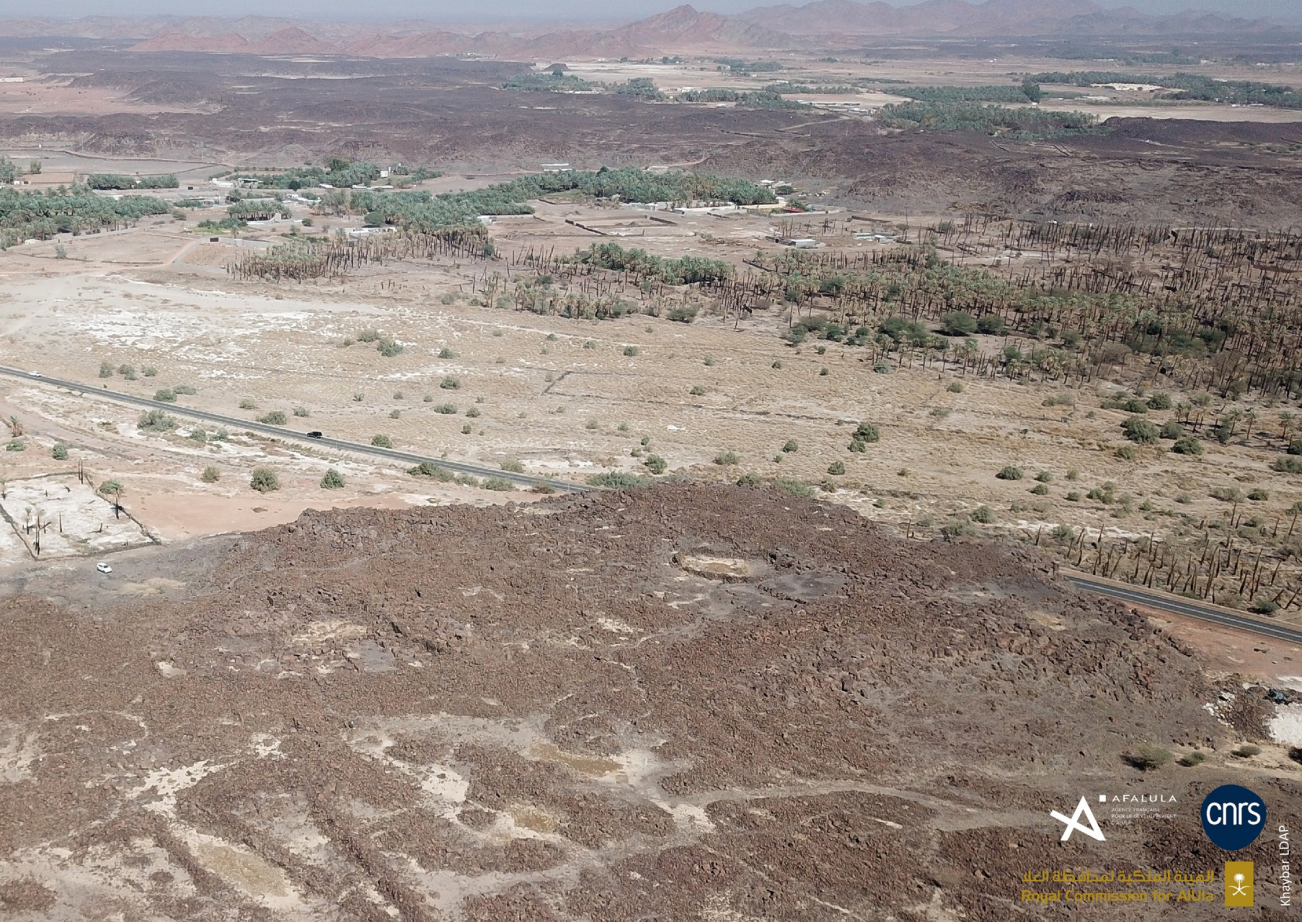
Al-Natah site in Khaybar Oasis.

The recent discovery of the al-Natah site in Khaybar Oasis (Fig 2), dated to the late Early Bronze Age (henceforth EB) and the Middle Bronze Age (henceforth MB), is a decisive step forward in our knowledge of the indigenous socio-political trajectory of Northwestern Arabia around 2000 BCE. Directly accessible below the surface there is almost a thousand years of use that for the first time reveals the morphology of a sedentary settlement linked to a rampart in the arid Northwestern Arabian desert between ca 2400 and 1500 BCE. This major discovery now makes it possible to consider the economic, political and social complexity of a sedentary settlement within a contemporaneous walled oasis ([33, 35]).

## Methods

### Context and survey

**Fig 3:**
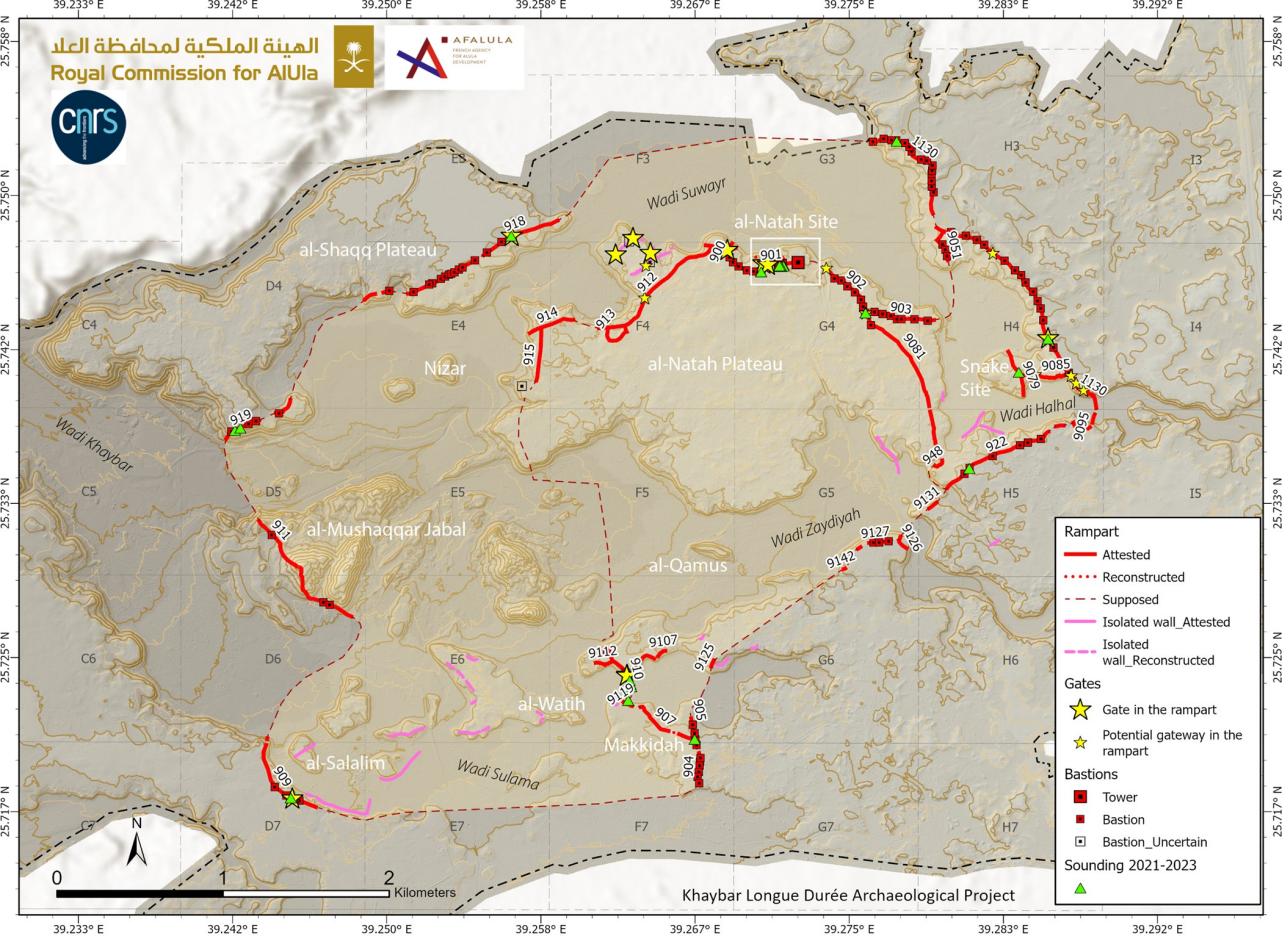
Map showing the location of the al-Natah site in Khaybar Oasis. Reprinted under a CC BY license, with permission from AFALULA-RCU-CNRS, 2024.

The oasis of Khaybar in Medinah province has been a major place of passage and sedentary life in the Saudi Hejaz desert since prehistory (Fig 1) ([40, 41]). Covering an area of roughly eight by seven kilometers, the oasis is today located inside the official jurisdiction zone of the Royal Commission for AlUla (henceforth RCU) which encloses an area of 56 sq. km, at the edge of a large lava field called Harrat Khaybar in Northwest Saudi Arabia. Situated in a hyper-arid environment, the oasis is formed by the confluence of three main wadis (al-Suwayr, al-Zaidiyyah and al-Sulama) leading to Wadi Khaybar and running between basalt plateaus and some rhyolite massifs. Springs found in these wadis allowed for the development of palm groves, agricultural areas, wells and farms of the Islamic period (Fig 3). Other archaeological vestiges (from all periods) are mostly located on the basalt plateaus, and are generally well-preserved notably notably because the modern city developed to the south of the RCU perimeter adjacent to the Qa‘ Qaran. Today, the oasis is a regional touristic center best known for the Battle of Khaybar in 628, and for its hilltop Islamic villages.

The Khaybar *Longue Durée* Archaeological Project (henceforth Khaybar LDAP, AFALULA-RCU-CNRS), started in 2020 and will end in late 2024. It aims to map and understand human occupation on a large geographical scale through time, in relation to the evolution of the oasis environment. The survey methodology of the RCU area is based upon a strategy of non-probabilistic and probabilistic survey samplings, combining remote sensing and the selection of field research areas for surveys and test excavations conducted by a team of more than 30 French and international permanent or short term contracted researchers. At this stage, Khaybar LDAP has recognized nearly 20,000 archaeological features, of which 6,000 have been described in the field.

**Fig 4:**
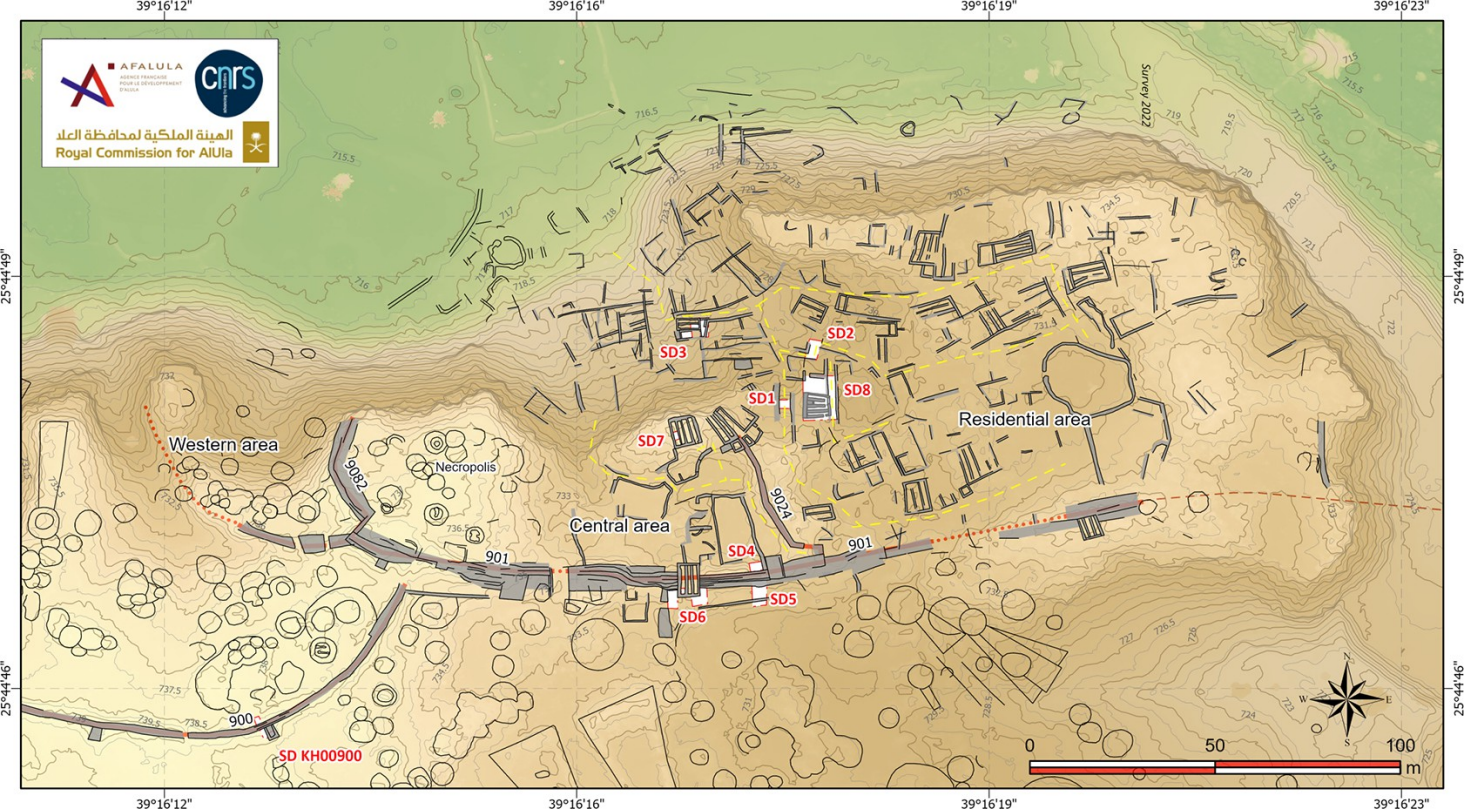
Map of the al-Natah site (KH02434), with plan of the architectural features identified on the surface (black lines; grey lines for reconstructions; dashed red line: Reconstructed layout of the rampart; numbers are written here without prefix KH for simplification).

**Fig 5:**
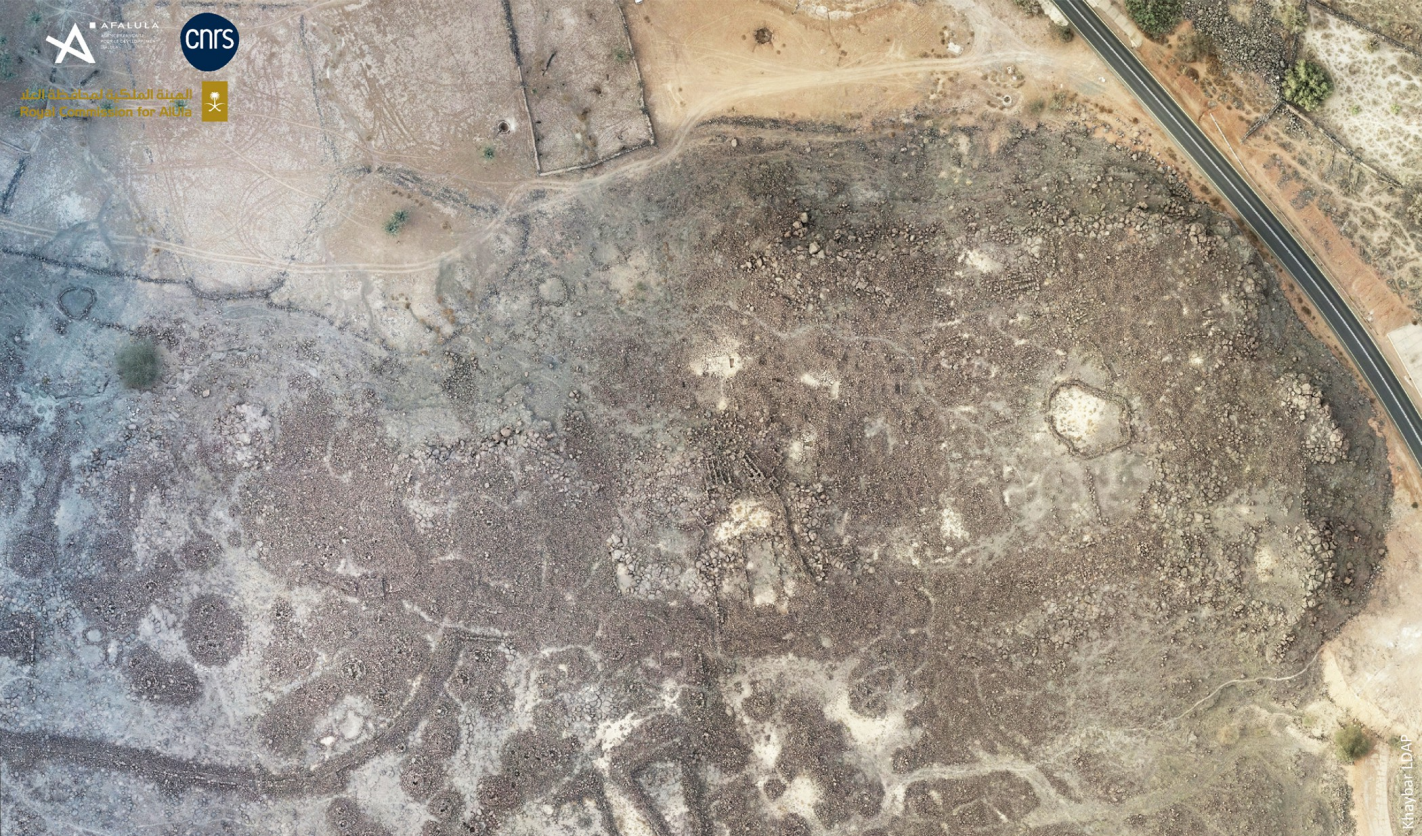
Orthophotogrammetry of the al-Natah site (KH02434). Reprinted under a CC BY license, with permission from AFALULA-RCU-CNRS, 2024.

The al-Natah site (KH02434) was identified during the first survey season of Khaybar LDAP in October 2020 and was studied and excavated until February 2024. Situated to the north of Khaybar Oasis, at a strategic point overlooking the 700-m-wide Wadi al-Suwayr (Fig 3), the site is now completely covered by a huge pile of basalt blocks from protohistoric remains—which partly explains why it was only discovered so recently. The structures are difficult to see, and tracing an overall plan was a complicated task, and only partially successful. In order to establish a coherent plan of the site without extensive excavations, which were beyond the scope of our project, a systematic survey was carried out in the field in March and November 2022, using high-resolution photographs produced by our project (1.5 cm/pixel on ©ARCGIS online project integrated into GPS tablets) (Figs 4 and 5).

In order to facilitate fieldwork and drawing, a virtual grid of 10 m squares had been set up during the previous season over the entire al-Natah site and the surrounding areas. Given the difficulties involved in identifying architectural structures among huge piles of stone blocks, only the walls and remains identified on the surface with a high degree of certainty (i.e. identified through aerial imagery and confirmed by field survey) were plotted on the site map: the lines of stone blocks observed in the field were drawn using polylines, and grey backgrounds were added to indicate widths. It was sometimes possible to reconstruct coherent architectural groups and units, despite considerable erosion of the site. The completion of the systematic field survey provides a good overall image of the site, its extent and organization, and also helps us to interpret the types of structures encountered. However, the site plan must be considered as preliminary until extensive excavations take place. The taphonomic conditions of such a site, set on a slope on basalt bedrock, are largely unfavorable to the preservation of architecture and continuous stratigraphy, with very little chance of recovering the levels of occupations that have been remobilized. With regard to the paleoenvironmental reconstruction of the al-Natah landscape, it should be noted that the neighboring Wadi Suwayr area has not yet been tested.

### Excavation

Alongside this survey, eight targeted soundings yielded archaeological material in context (pottery, grinding tools, fauna bones, beads, metal fragments) and provided valuable information about the type and chronology of site development (dwellings, streets and ramparts) (Fig 4). The rich dump layers from sounding 3 (KH02434.300) were all sieved with a 5-mm mesh, while samples were selected for archaeobotanical and environmental DNA analyses carried out in France. Finally, three soundings (3, 5, 6) were analyzed for sedimentary deposits by geomorphologists. During survey and excavations, more than 6000 pottery sherds were collected at the al-Natah site and compared with assemblages from other parts of the oasis ([42, 43]).

## Results

### Site layout

The al-Natah site (KH02434) covers approximately 2.5 ha (max. length 250 x max width 130 m), mainly on the high and flat parts of the edge of al-Natah plateau at an average elevation above sea level of 730 m, as well as a ridge and its slope at the northern edge of the site. The settlement extends down the slope to an elevation of 718 m towards the cultivated areas of the valley. Farther south, on the same plateau, a dense concentration of protohistoric tombs, in particular the famous funerary avenues, were identified ([40, 44]).

The settlement controls two major communication routes: the wadi itself along an east-west axis and a section of the road leading to Tayma and perhaps AlUla to the north and Medinah to the south. This route is now delineated by the modern asphalt road on the eastern side of the site, that runs in a natural depression of the plateau. Today the valley is covered by abandoned cultivation plots and a few farms at an average elevation of 710–715 m. Many wells and water sources were identified in the sector, including three wells at the base of the cliff in the vicinity of the site. There was thus a good water supply in the sector.

**Fig 6:**
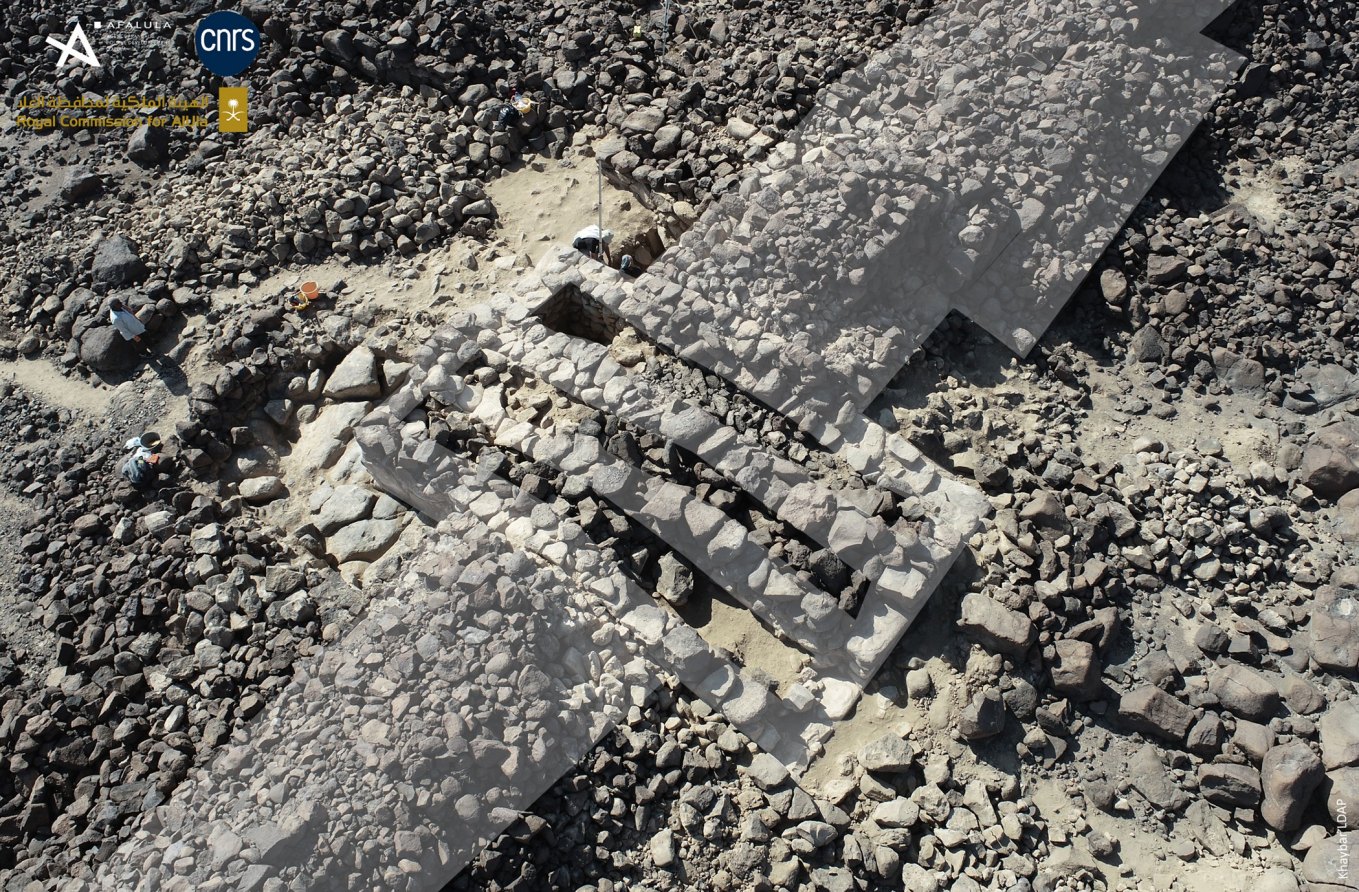
A tower in the southern rampart of al-Natah site, facing southwest. Reprinted under a CC BY license, with permission from AFALULA-RCU-CNRS, 2024.

The southern boundary of the site is demarcated by rampart KH00901 (and KH09082). The impressive thickness of this dry-stone structure, ranging between 3.5 m to perhaps 6 m wide, and the presence of two large towers (Fig 6), clearly distinguish it from other enclosure walls with bastions, in particular the nearby 2.1 m-thick walls KH00902 and KH00900. Unfortunately, this rampart KH00900 is covered by a mass of collapsed basalt blocks. In addition to multiple episodes of masonry dismantling, the presence of many earlier and later stone structures greatly complicates the task of reconstructing the rampart layout. When no facing is visible, only the existence of stone mounds can indicate the presence, or disappearance of the rampart at its eastern and western ends. For all these reasons, the question of access to the site from the south was not resolved.

We documented two main areas circumscribed to the north of rampart KH00901, and spatially subdivided by rampart KH09024: 1. a residential area in the east, 2. a central area with a necropolis in the west. A third area, with a double entrance (?), was identified on the western side of the site (Western area). The presence of sometimes well-preserved stone circles in the lower part of the village and an Islamic inscription in the central area, points to later ephemeral use of al-Natah site, although no diagnostic Islamic pottery was found there.

#### The residential area

**Fig 7:**
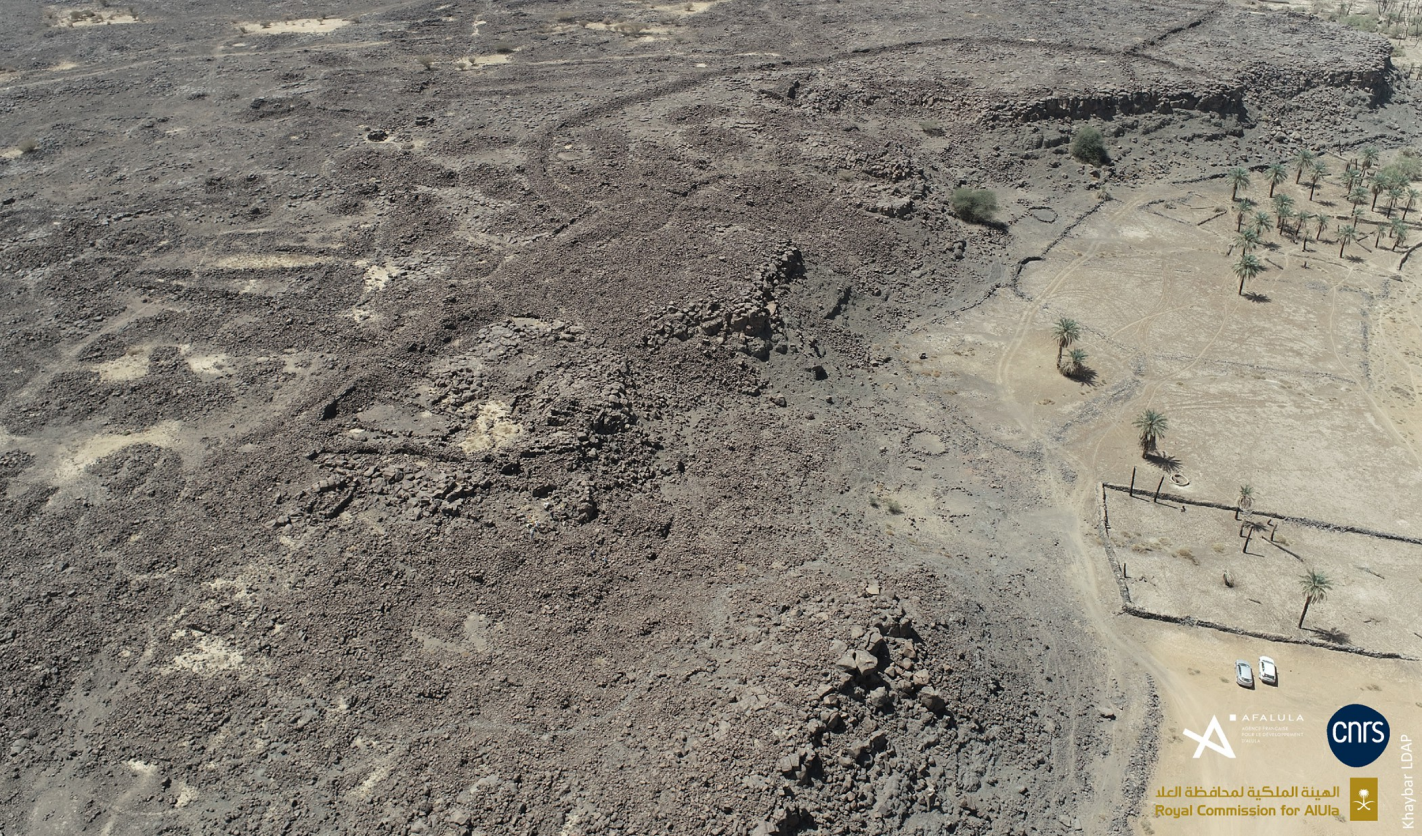
The residential area of the al-Natah site, looking west.

The eastern sector of the al-Natah site, which, unlike the other sectors, yielded large quantities of domestic material (pottery and grinding stones) on the surface, was certainly a residential area (Fig 7). It is composed of a series of (at least 50, perhaps up to 70) isolated or connected elongated quadrangular units which constituted the basement of probably tall (possibly earthen) now collapsed dwellings. These units were accessed along by curvilinear 2-m wide streets or narrower alleys. Most of these dwellings are situated in the lower parts of the site, particularly in the bottleneck leading to the wadi, or in high positions on the northeast cliff. There was a general tendency to build rectilinear walls, but several remains show irregular and curvilinear boundaries. Although most of the structures were roughly oriented north-south, others were offset from this axis, adapting to the terrain rather than adhering to strict overall planning. Topography thus played a decisive role in the siting of structures, as can be unambiguously seen around and along the small ridge on the northeastern side of the site.

**Fig 8:**
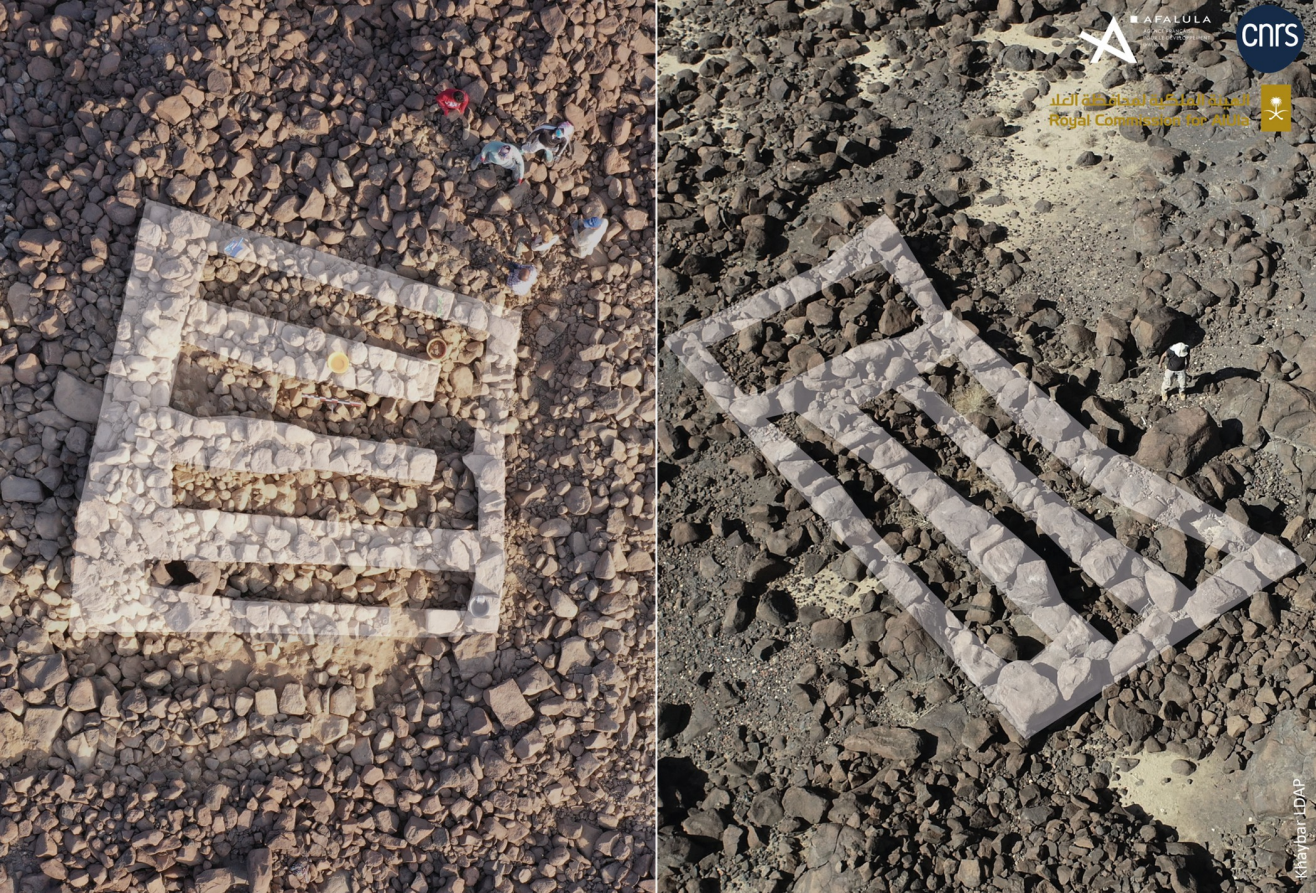
A quadripartite unit (sounding 8) during excavation (left, looking north) and a tripartite dwelling with an added platform(?) on its smaller side (looking north-west) at the al-Natah site. Reprinted under a CC BY license, with permission from AFALULA-RCU-CNRS, 2024.

The architectural units seem to follow a standardized architectural pattern and to use similar construction techniques. They generally appear to be formed by a juxtaposition of two to four rows or corridors of varying dimensions, about 0.6 to 1.1 m wide (often 0.8 m) and between 4.5 and 8.5 m long (Fig 8). The spaces are defined by thin walls, usually between 0.5 and 1 m thick. When they can be confidently identified, the internal subdividing walls are often narrower than the external walls. The masonry of the exterior faces of the units appears to be better built and more regular than their internal faces–which were probably not designed to be seen, as confirmed by the gradual accumulation of sediments and fallen basalt blocks and the absence of windows. The presence of a doorway and a passage in one of the corridors of large buildings is however not ruled out, and makes it possible to envisage interior access (by ladders?). Sometimes a quadrangular space appears to abut the front of this type of rectangular unit, possibly indicating the presence of a platform with a staircase.

**Fig 9:**
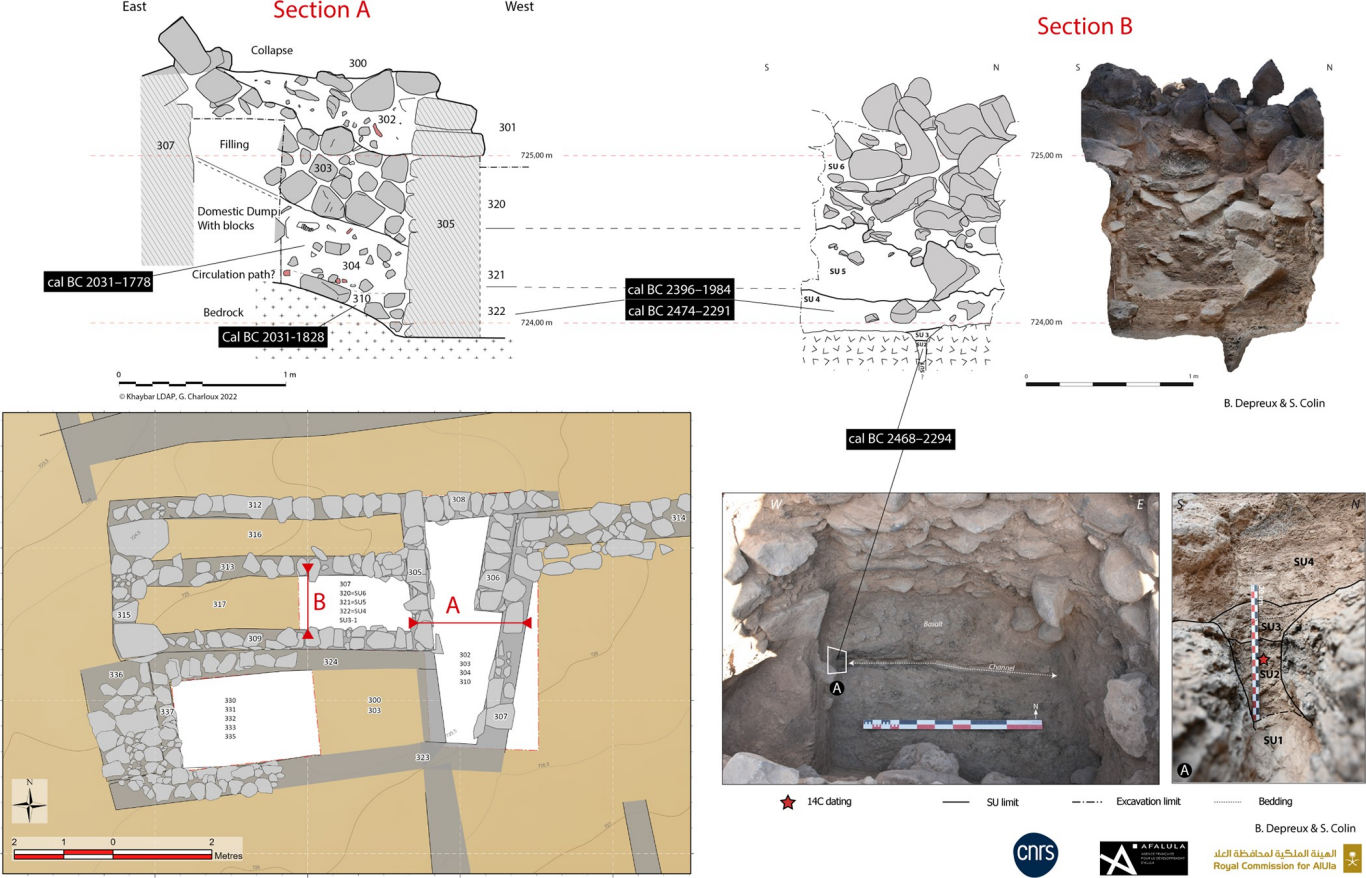
Sounding 3, plan and archaeological sections A & B with SU numbers and location of ^14^C datings (Section A in SD3a, Section B in SD3b). Reprinted under a CC BY license, with permission from AFALULA-RCU-CNRS, 2024.

Five soundings (henceforth SD) were excavated in the residential area of the site (SD1-3 & 7–8). Soundings 3, 7 and 8 focused on bi- and quadripartite units while SD1 and 2 focused on streets. The most interesting, SD3, revealed two different contexts, inside and outside a small bipartite building located on the slope of the basalt plateau (Fig 9). The interior sounding (SD3b) revealed a fill accumulated though a succession of flows (SU4-5; SU = Stratigraphic Unit), the competence of which gradually intensifies towards the top, with the interior part of the building appearing to function as a trap for sediments capturing run off deposits along the slope. The top of the fill is marked by the collapse of the adjacent walls (SU6). These features suggest that the interior space was at least partially empty when the building was in use. Beneath this fill, a linear hollow in the substrate, oriented in the direction of the slope, could be interpreted as a small drainage channel, filled with fine, compact, cracked sediments containing fragments of bone and pottery (SU1-3). It seems that this interior space was mainly used as a storage area (or other undetermined function), before being progressively filled in by trapped sediments and then by the collapse of the building’s walls. The dating carried out on a bone collected from the fill of the channel (SU2) gives an age to the construction of the building of between 2468 and 2294 BCE (UGAMS-65431, Table 1). Two other dates (DeA-43555 and DeA-42272) from charcoal from the first layer filling the space inside the building (SU4/322 = KH02434.322), give a more imprecise range of between 2474 and 1984 BCE, posterior to the construction of the walls. This evidence testifies to an early occupation phase of the al-Natah site in the second half of the third millennium BCE.

**Table 1:** ^14^C dates from the al-Natah site (date in italics is rejected due to incomplete treatment of the sample).

Lab no.	Sample ID	Material	Age ^14^C BP	Age cal. BCE (2σ)
DeA-43555 (n2)	KH02434.322 (SD3b)	charcoal	3917 ± 31	2474–2291
UGAMS-65431	KH02434 SD3b SU2	Bone (bioapatite)	3900 ± 30	2468–2294
DeA-42272 (n1)	KH02434.322 (SD3b)	charcoal	3761 ± 52	2396–1984
DeA-43996	KH02434 SD6c n°2	charcoal	3727 ± 30	2269–2031
BETA-625967	KH02434.310 (SD3a)	charcoal	3590 ± 30	2031–1828
DeA-43980	KH02434.304 (SD3a)	charcoal	3581 ± 35	2031–1778
DeA-43993	KH02434 SD6b n°1	charcoal	3572 ± 31	2025–1777
DeA-43994	KH02434 SD6b n°6	charcoal	3525 ± 35	1946–1746
DeA-39461	KH02434.406 (SD4)	charcoal	3447 ± 30	1880–1642
DeA-43995	KH02434 SD6b n°7	charcoal	3427 ± 33	1875–1624
BETA-625966	KH02434.405 (SD4)	charcoal	3420 ± 30	1804–1623
DeA-39460	KH02434.010 (SD1)	charcoal	3410 ± 31	1871–1617
DeA-39462	KH02434.210 (SD2)	charcoal	3382 ± 38	1863–1540
DeA-42275	KH02434.622 (SD6c)	charcoal	3209 ± 34	1531–1418
DeA-43987	KH02434 SD6a Log.1 n°1	charcoal	3182 ± 29	1504–1412
*DeA-44001	*KH02434 SD6b n°4*	*charcoal*	*3160 ± 36*	*1504–1314*
DeA-44320	KH02434 SD6a Log.3 n°2	charcoal	3075 ± 70	1497–1126

A sounding outside the building (SD3a) revealed a rich dumping context (KH02434.304-KH02434.310), filling a space blocked by the addition of later walls. This dating indicates a phase of occupation of al-Natah site in the first half of the second millennium BCE, confirmed by two charcoal samples from streets bordered by walls in soundings 1 and 2 (KH02434.010 and KH02434.210), possibly showing occupation until the mid-16th century BCE.

#### The central area

**Fig 10:**
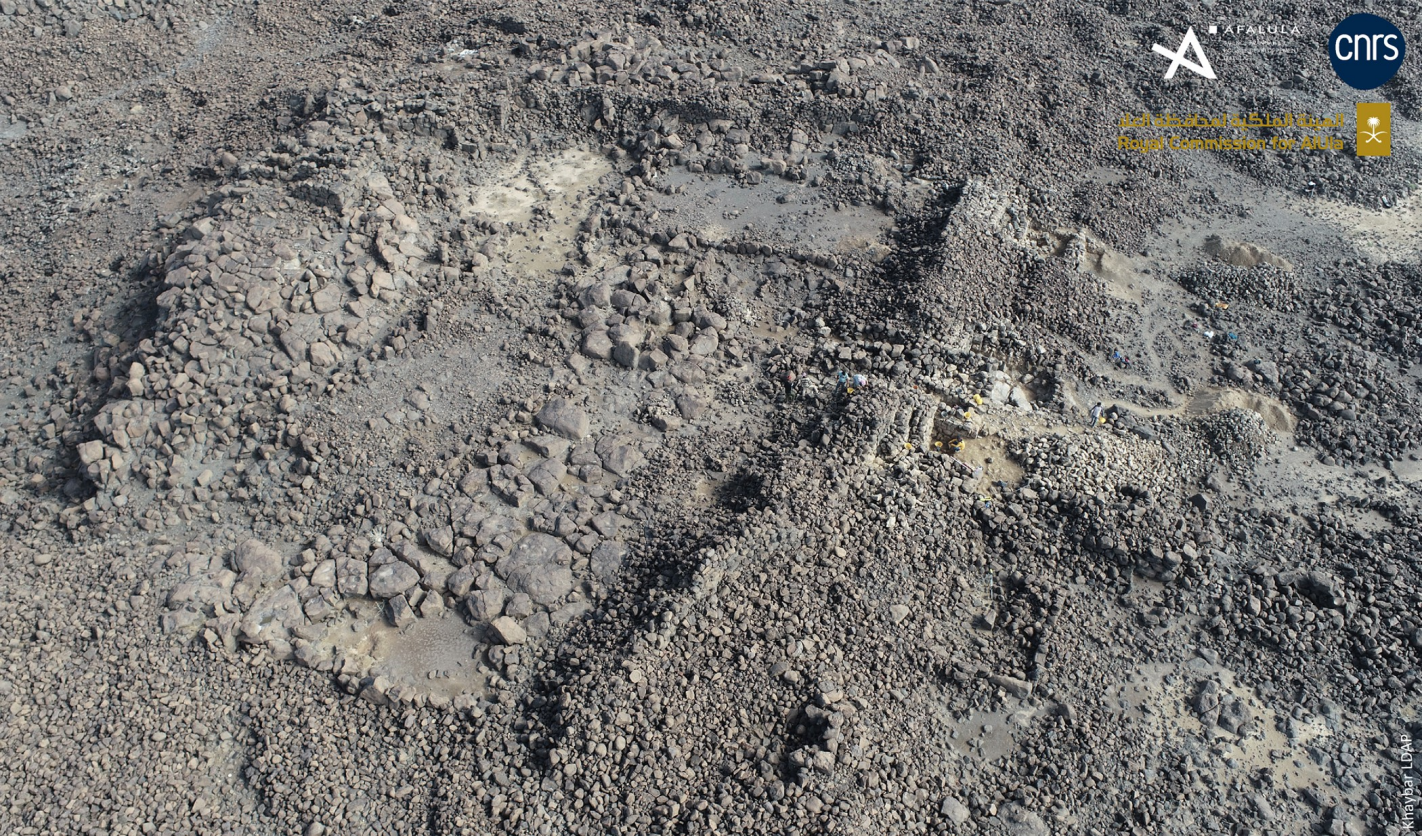
The central sector of the al-Natah site.

The central sector is defined by ramparts KH09024 to the east, KH00901 to the south and KH09082 to the west. Unlike the residential area, this sector–which is slightly more elevated than the nearby settlement–appears today to be heavily leached and eroded (Fig 10).

A north-south subdividing wall was located west of the central area, but this narrow wall was certainly not comparable to KH09024. A clear functional distinction is visible on either side of the southern boundary of this narrow wall, but this may result from the numerous disturbances and redevelopments towards the west over time. The examination of the western part of the central area revealed a necropolis, contemporaneous with the al-Natah site in the first half of the second millennium BCE. This necropolis is made up of a unique type of megalithic tombs, the “stepped tower-tombs”−a new type in Arabia, defined as a large and high circular tomb with exterior stepped walls and a burial chamber with internal pillars−arranged in isolation or as an aggregate. They appear more recent than the tailed tombs found in the funerary avenues. The avenues and the tailed tombs are cut by rampart KH00900, dated to the first third of the second millennium BC, and abutting against al-Natah rampart KH00901, to the west of the town.

In the eastern part of the central area, the two fairly well-preserved quadripartite buildings to the north–comparable to those in the residential area in terms of their distinctive layout with narrow, parallel, contiguous rows − located on high ground and with a wide view over the surrounding area, were probably the dominant strategic and political center for the whole site.

To the south of these buildings, a small central depression or courtyard is delimited by trapezoidal zones bounded by alignments of very large blocks. These alignments could constitute the last remains of ancient monumental trapezoidal tombs, in the line of a southwest/northeast funerary avenue, reused in the Middle Bronze Age. Corridors demarcated by open-air structures, appear to regulate circulation, especially towards the small central square.

**Fig 11:**
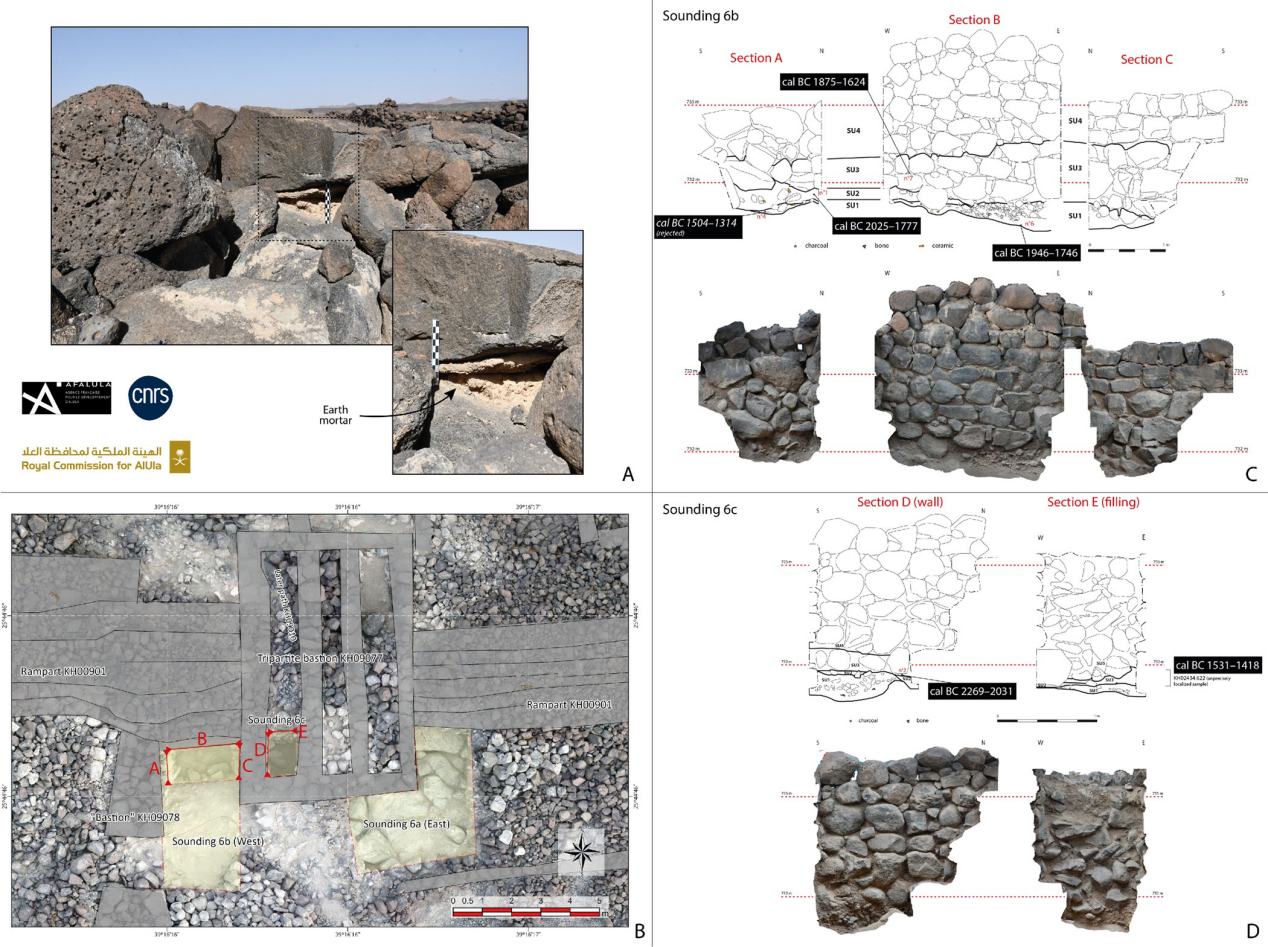
The rampart of al-Natah: A. Traces of mudbrick in the masonry; B. Plan of soundings 6a-c in and around tripartite tower KH09077; C. Sections in sounding 6b; D. Sections in sounding 6c. Description of the layers (SD6b-c): the grey sandy-gravelly basal unit SU1 is the first unit lying on the basaltic substratum. No weathering layer of the substratum is observed. The presence of pebble beds of varied lithology within an otherwise fairly homogeneous unit indicates the probably anthropogenic origin of this deposit, which could not be transported by runoff. A dark brown anthropogenic paleosoil with granular structure, composed of ceramic sherds, bones and numerous charcoal pieces, was identified and described (SU2). Its presence is discontinuous: it is well developed to the west of SD6b, where it is beveled under SU3, and present in the form of a thin partial layer in SD6c. This layer could be evidence of a very local reworking of the occupation levels at the site. Both layers seem to predate the construction of the bastion and the rampart, but could also constitute pre-construction preparation. SU4 corresponds to run-off facies that fill in the gaps between the blocks of the built elements. The concordance of the sedimentary facies and geometry of the units located between the masonry blocks of the structures (SU 3 and 4), suggests that the deposition of SU4 results from the same sedimentary process, probably post-construction. We assume here that these runoff facies mark the very local movement of material eroded from the top of the dismantled structures, filling the partially empty spaces between the blocks. This raises questions about the construction method, which potentially combines dry stone construction with the use of raw earth. Finally, in SD6c, SU5 is mainly composed of blocks with a sandy matrix marked by these runoff facies which fill the gaps between the blocks. It constitutes a deliberate filling of the tripartite bastion, with probable collapse of the wall, and marks the end of the use of the structure. Reprinted under a CC BY license, with permission from AFALULA-RCU-CNRS, 2024.

The excavation of three soundings (SD4 to 6) along the rampart seems to have established contemporaneity between the rampart and the al-Natah residential area. On architectural grounds, the two large tripartite towers integrated into the rampart technically and morphologically correspond to the dwelling units in the residential area (compare Figs 6, 8 and 9). Sounding 6, the most relevant, covered both the interior of the easternmost tower (SD6c) and the southern face of the rampart on either side of the tower (SD6b and 6a) (Fig 11). The oldest context corresponds to the base of the interior space of the tower, where the contact between SU2 and SU3 is dated to 2269–2031 BCE (DeA-43996). This is a similar date to the bipartite unit in the residential area (SD3a), and shows that this tower later integrated into the rampart, may have served as a dwelling in a first phase. A sample imprecisely located on top of SU3 was dated to 1531–1418 BCE (DeA-42275), and therefore provides a *terminus post quem* for the fill with its numerous joined blocks in a sandy matrix.

The results obtained from layers below and abutting the rampart in SD6b and SD4 (rampart named Gr.A in [35]) confirm that it was established in the first half of the second millennium BCE. SD6b provided three radiocarbon dates from charcoal, collected from the preparatory layers and in the earth mortar of the rampart facing (SU1-3). These dates are close, partially overlapping, and provide a chronological interval for the construction of the rampart between 2025 and 1624 BCE (Table 1). SD4, established against the north face of the rampart, revealed several layers of occupation, adjacent to the rampart, the most recent of which is dated to 1880–1623 BCE (BETA-625966, Table 1). Finally, SD6a provided two dates: 1504–1412 BCE (DeA-43987) at the base of a potential additional wall of the rampart, and 1497–1126 BCE (DeA-44320) at the base of the rampart collapse. They reveal a *terminus post quem* from the late 16th-14th centuries BCE, but they seem to point to a late restructuring of the fortifications with the addition of a reinforcing wall, which would explain the impressive thickness and build-up of wall masonry, and/or to a phase of abandonment of the site.

### Bayesian modelling of radiocarbon dates

Based on the previous description, the architecture at the al-Natah site appears to be rather standardized and seems to correspond to a main period of occupation, although in-site spatial reorganization was identified because of the blocking of a small street with the dumping of fill in SD3. Radiocarbon datings and geomorphological analyses also confirm a long use of the site during the Early and Middle Bronze Ages, but with possible disruptions. The absence of preserved occupation levels makes it difficult to establish the occupation sequence of the site. It was determined by the radiocarbon dating of 16 charcoal samples and one bone sample (Table 1). All these radiocarbon-derived ages were calibrated using IntCal20 ([45]). One was rejected (DeA-44001) due to incomplete treatment of the sample, only a gentle acid preparation with one-step combustion was carried out, which may affect the age since no soluble humic acids were removed. From a relative dating perspective, the pottery found in a sealed context provides a rough but consistent timescale. The chronological sequence is based on the examination of civil or monumental contexts that were not affected by late (probably Islamic) redevelopment in the south-eastern part of the residential sector.

**Fig 12:**
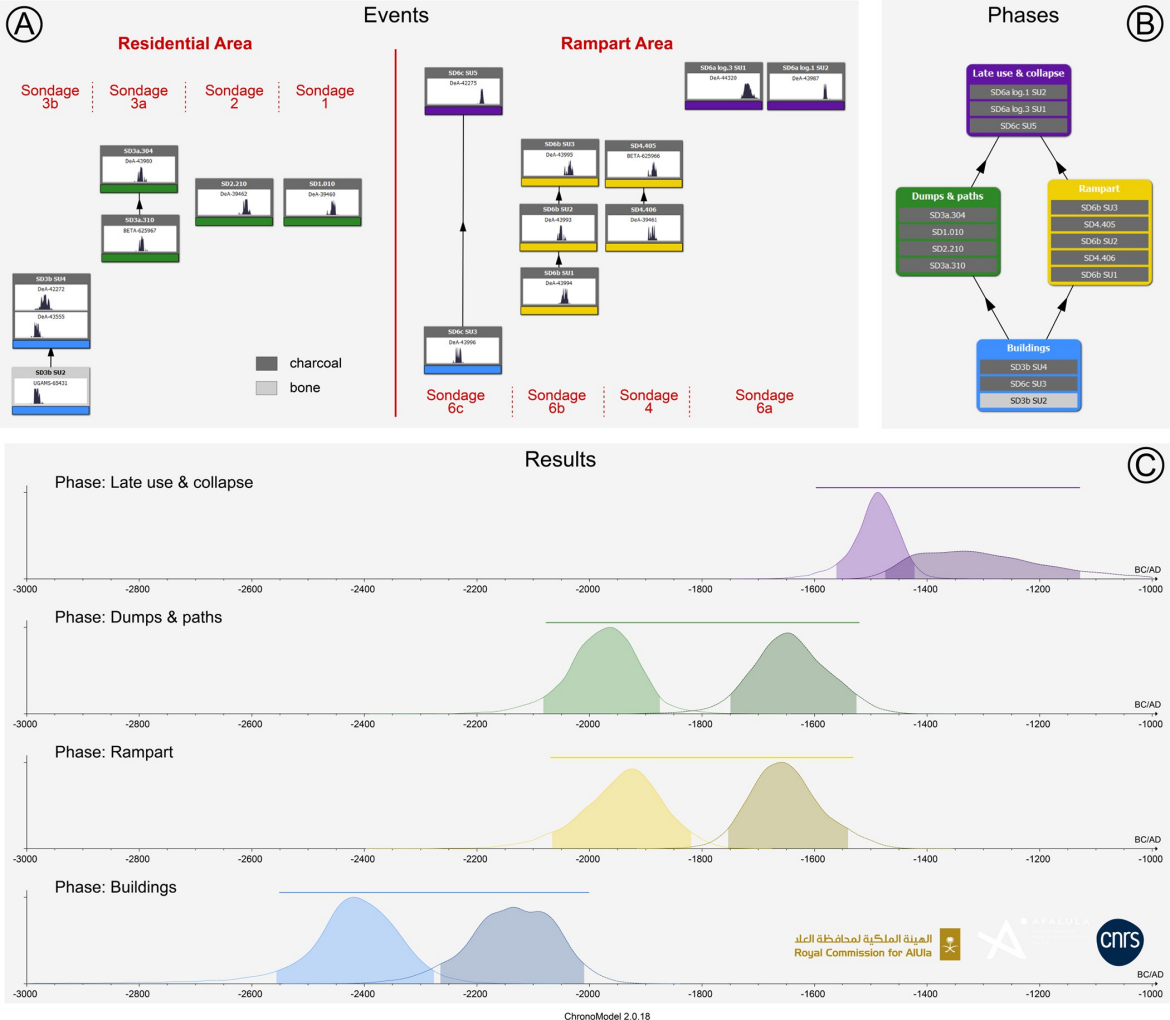
Schematic representation of the al-Natah Bayesian model in ChronoModel V2.0.18: A. set of dated "events" between which stratigraphic constraints can be applied (arrows), B. grouped by phase for which a succession constraint is applied, C. a posteriori phase start and end distributions and 90% phase time range (horizontal bar above the two distributions). Reprinted under a CC BY license, with permission from AFALULA-RCU-CNRS, 2024.

The ^14^C dates ("events") were subjected to Bayesian analysis using ChronoModel V2.0.18 software ([46]), enabling a chronological model of the al-Natah site to be produced in which the stratigraphic data of relative chronology and archaeological contextualization were integrated (Fig 12). Four groups ("phases") were thus identified: the interior spaces of the bi- and tripartite architectural units, the exterior spaces of the residential area, the layers of occupation or construction of the rampart, and the levels of backfilling or reuse.

The earliest dates found on the site come from the interior spaces of the bi- and tripartite units, whether in the residential area or integrated into the rampart (Fig 12). It would seem, therefore, that the first period of occupation of the site, characterized by the construction of these buildings with their specific architecture, was quite early, taking place between 2419 BCE and 2135 BCE (Table 2). This was followed by a second period of occupation, marked by the contemporaneous construction and use of the rampart, with the dumping of domestic deposits and the use of circulation spaces in the residential area. This period spans from ca. 1964 BCE to ca. 1647 BCE (Table 2). Finally, a last period has been identified, marked only by infilling, collapse or re-use of the rampart. Beginning around 1488 BCE, the end, which is less precise due to the small number of dates associated with high margins of error, is dated to ca. 1334 BCE (Table 2). However, these dates push back the occupation of the site by three centuries, perhaps as far as the 14th century BCE, for contexts absent in the residential area. This could be evidence of a late redevelopment of the fortifications, in particular with the addition of a reinforcing wall. These data raise the question of whether the occupation of the al-Natah site was continuous or interrupted. As things stand, although they are distinct, these three periods could just as well indicate a continuous occupation. In the absence of further available information, we will refer to them as early, middle and late occupations.

**Table 2:** Results of Bayesian modelling in ChronoModel V2.0.18, calibrated in BCE with Intcal20. HPD, Highest Probability Density region at 90% for phase start and end dates; MAP, Mode a posteriori for phase start and end dates.

Phase	MAP	HPD start	HPD end
Buildings—Begin	-2419	-2556	-2276
Buildings—End	-2135	-2265	-2009
Rampart—Begin	-1924	-2066	-1819
Rampart—End	-1659	-1754	-1541
Dumps & streets—Begin	-1964	-2082	-1875
Dumps & streets—End	-1647	-1750	-1526
Late use & collapse—Begin	-1488	-1561	-1422
Late use & collapse—End	-1334	-1474	-1128

#### The al-Natah site in its local context

**Fig 13:**
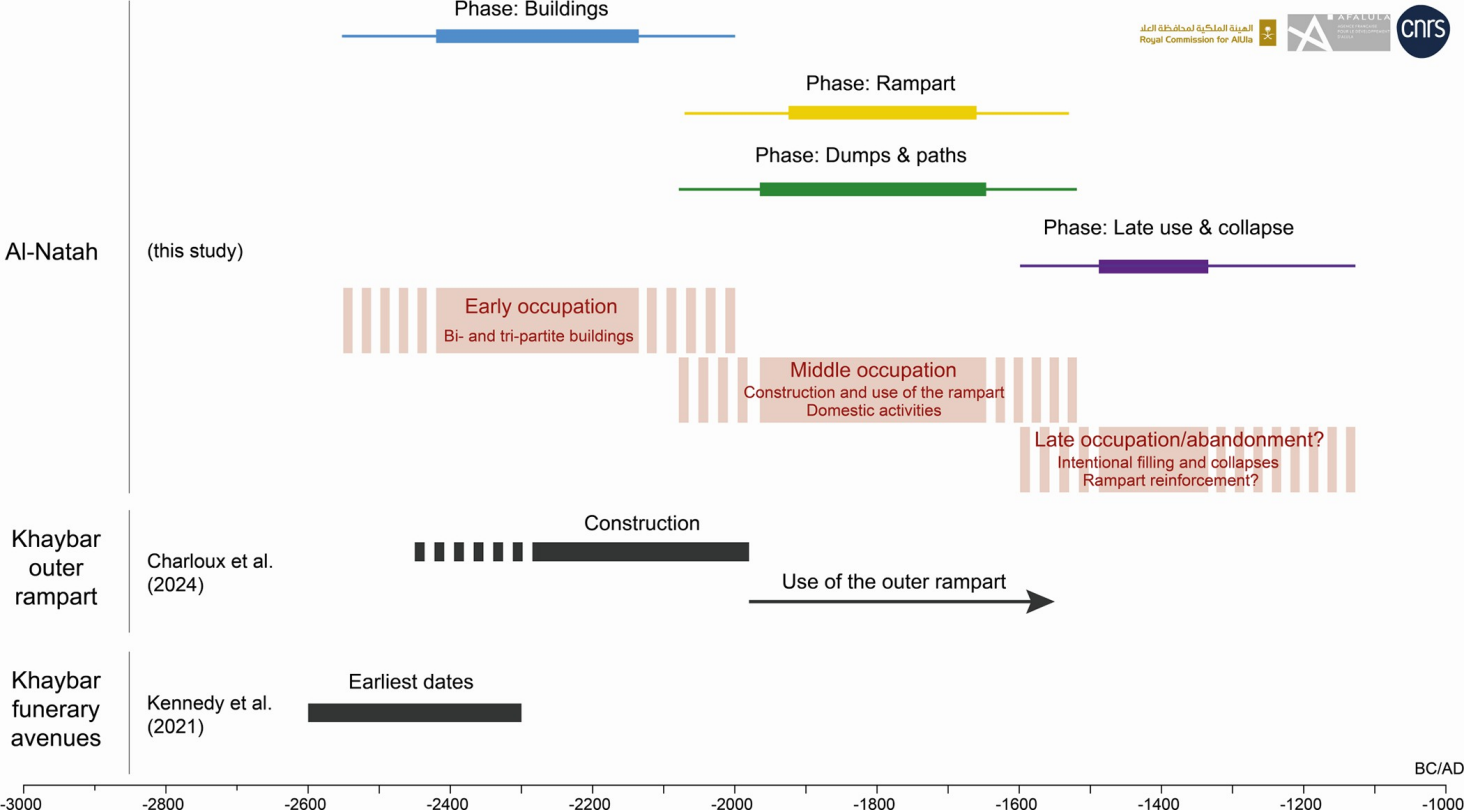
Bronze Age archaeological occupations in the Khaybar oasis, Khaybar LDAP. Reprinted under a CC BY license, with permission from AFALULA-RCU-CNRS, 2024.

To sum up, the ^14^C data confirm a long-term sedentary settlement at the al-Natah site as early as 2419 BCE (2556 BCE if the high margin of error is taken) (Fig 13). This early occupation, characterized by the construction of bi-, tri- and quadri-partite buildings, is partly contemporary with the development of the funerary avenues of tailed tombs, which the earliest ^14^C results date to between 2600 and 2300 BCE ([40]). It is interesting to note that the funerary avenues in this part of the plateau are all directed towards the site of al-Natah, which seems to constitute a node in the local territorial network, raising questions about the spatial relationship between the funerary and settlement zones. Moreover, this early occupation is also partly contemporary with the period of construction of the outer rampart of the oasis, dated to between 2250 and 1950 BCE ([35]). This supports the idea of a sedentarization process by mobile groups during the second half of the third millennium BCE, which is also marked by a drier period due to the 4.2-ka climatic event ([47–49]). The need to fortify a territory with the creation of the walled oasis thus arose at a time of climatic oscillation and possible regional social tension, after a first stage of communal agglomeration of nomadic groups around ecological niches visible through the creation of vast funerary avenues connecting the oases ([40, 44]). During this second stage, in the second half of the third millennium BCE, funerary avenues, although cut or reused by the ramparts at Khaybar, probably continued to function together with the sedentary settlement at al-Natah.

The site of al-Natah prospered in the next phase at the beginning of the second millennium BCE. This middle occupation was characterized by the construction and use of the rampart and by intense domestic activity in the residential area in the 20th-16th centuries BCE, probably in a slightly more favorable paleoclimatic context ([48, 50, 51]). Interestingly, this phase followed the period of construction of the outer rampart and was contemporary with its use (Fig 13). This shows that the fortification of the site was undertaken after the vast work of fortifying the oasis and that these different stages could be part of the same trajectory of sedentarization.

Settlement at the site lasted until at least 1500 BCE and possibly until 1300 BCE, according to radiocarbon dating (Fig 13). However, these dates only apply to situations where structures were deliberately filled in or collapsed, and where the rampart was probably reinforced. The absence of Late Bronze Age Qurayyah Painted Ware from the site could either suggest that the production lies out of the distribution range or, more likely, that the main period of activity at the al-Natah site preceded QPW. In the absence of more recent material, the reasons for the abandonment of the site are still enigmatic: return to nomadic life, disease, climate deterioration, etc.

### Site morphology and function

In order to better characterize site morphology and function with a view to evaluating the place of the site in regional political development, several criteria are assessed below in a preliminary manner: population and landscape, function, economy, contacts and social complexity.

#### Estimated population of the al-Natah site

**Fig 14:**
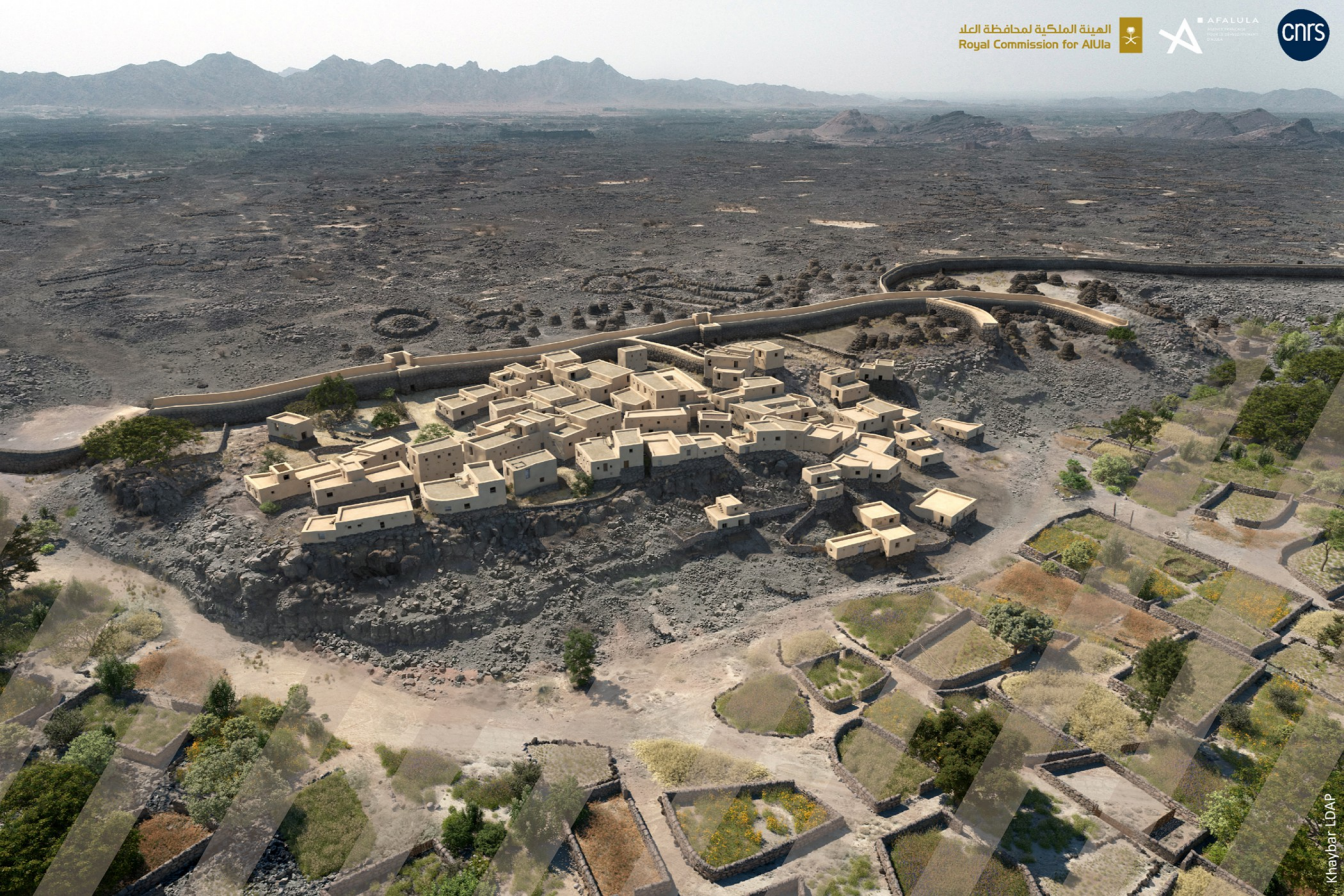
3D virtual reconstruction of the Bronze Age site of al-Natah. Reprinted under a CC BY license, with permission from AFALULA-RCU-CNRS, 2024.

Estimating the number of inhabitants can yield a more accurate idea of site function. However, this is a hypothetical approach, particularly in the absence of building elevations. Based on surveys, our current estimate points to around 50 dwellings, concentrated in the eastern sector. As the south-eastern part was dismantled for more recent buildings, this estimate could rise to around 55–70 dwellings. The foundations of the houses (approx. 5 x 10 m for a tripartite house, 7 x 8 m for a quadripartite unit) were probably used for storage or as a crawl space according to the geomorphological results, while the living spaces would have been located higher up. Given the thickness and configuration of the structures, it is reasonable to assume that they were two and sometimes possibly three floors high, giving a total height of around 8 m, including the foundations (Fig 14). This type of construction is reminiscent of traditional Arabian tower houses, particularly the much later ones in the Najd region [52] and in the 20th-century Khaybar oasis, or the three to four floors high tower houses at antique Qaryat al-Faw (a far cry from reconstructions of the nine-floor, 30-m-high buildings at Shibam, Yemen [53]). The number of inhabitants in the Yemeni tower houses comprised a single family of around six to 20 inhabitants, unlike certain Egyptian sites which brought together several family groups and therefore probably more individuals ([53]: 16). On this basis, and assuming that each dwelling housed an extended family comprising an average of around ten individuals, we can envisage a population of around 500 people in the residential area (1.6 ha), which amounts to an approximate density of 312 inhabitants/ha, as an indicative range. By way of comparison, Zorn ([54]) inferred a density of 450 inhabitants/ha for the small fortified site of Tell en-Nasbeh in the southern Levant. Broshi and Gophna expect an average density of 150 to 250 people/hectare ([55]). Wilson points out that most studies of population density in ancient towns suggest a ratio of between 100 and 400 inhabitants/ha ([56]: 171). However, the different types of town plans imply huge demographic variations.

#### The walled oasis landscape

The defensive organization of the al-Natah site, with a thick southern rampart and the absence of protection on the northern side in Wadi al-Suwayr, implies that the vast 14.5 km long outer rampart of the oasis not only protected the agricultural areas, but also the site itself, and potentially other settlements ([35]). The al-Natah rampart, on a secondary inner line of rampart, was only built during the middle phase (Fig 13). Given the location, size, chronological sequence, the absence of any other major contemporaneous settlement found in the oasis, and its protection by a line of ramparts with bastions joining the contemporaneous outer rampart, the al-Natah site seems to have formed the heart of the fortified oasis and very probably its political center.

The impressive extent of the walled oasis (1100 ha), in comparison to the limited surface area of the al-Natah site, nevertheless raises questions about human settlement in the oasis. Despite heavy disturbance (notably the construction of *marabid*, circular constructions established by the Bedouin populations and modern "fortresses") and the absence of sedimentation, several strategic locations yielded a high density of Late EB-MB Red Burnished Ware on the surface, notably the Snake Site, al-Watih, Makidah, Nizar, and al-Salalim ([57]). Some of these sites were protected by dedicated curtain walls, yet none of them has yielded traces of civil architecture from this period. It may be more pertinent to consider a multi-scale and multi-type settlement, with a central fortified settlement (al-Natah) and ephemeral camps. In fact, it is hard to believe that all the remains and dwellings in this area have completely disappeared while ramparts and some of the earlier structures have partially survived. Indeed, funerary avenues and ancient tombs have been preserved on some of these sites, despite modern construction, such as at Makidah (Fig 4). However, we must remain cautious in our interpretations. As was observed in the soundings excavated in the modern villages of al-Watih, Qamus, and Nizar, the absence of older remains is suspect, especially as ancient occupation cannot be refuted, given the late pre-Islamic history of local fortresses and the occasional presence of Thamudic inscriptions. We might, therefore, suggest that the remote locations of the abovementioned sites were allocated by a central authority to mobile groups of desert pastoralists. The entire oasis could have provided a more or less ephemeral area of security and refuge, particularly during the summer seasons in this hyper-arid region. These areas of protected exchange with al-Natah allied groups could have provided a complementary food supply and security, close to water sources.

#### Mutations in the funerary landscape

Between the middle of the third millennium and the first half of the second millennium BCE, the funerary landscape of the Khaybar oasis has also undergone profound changes, in line with the progressive sedentarization of the population groups in the al-Natah area. In the second half of the third millennium BCE, funerary avenues of tailed tombs dated between 2600 and 2300 BCE [40] materialized routes between regional oases [44]. The radial organization of the funerary avenues around the regional oases testifies to the grouping of population around well-watered landscapes [40]. In the Khaybar oasis, the construction of the various types of tailed tombs and their location in the avenues seem to have been governed by specific rules, reflecting a collective vision of the space occupied. Survey of the ramparts showed that these funerary avenues were systematically integrated in or cut by the inner and outer walls in the oasis, as in al-Natah. However, although the tailed tombs seemed not to have been built later, the funerary avenues were very probably still used in MB-LB periods, as shown by the late reopening of these tombs. Tower-tombs and tailed tombs within the funerary avenues progressively gave way to the construction of late third-early second millennium BCE stepped tower-tombs near al-Natah. Therefore, whereas burials were previously distributed along the funerary avenues and occurred across the area of the oasis, stepped tower-tombs were then grouped together close to the site within the ramparts of the walled oasis, creating a veritable necropolis. This grouping of graves confirms a change in burial practices linked to the nearby sedentary settlement. Excavations of funerary monuments point to similar conclusions: ceramic vessels are omnipresent in the stepped tower-tombs of the necropolis, while they are rare or non-existent in the tailed and tower-tombs of the funerary avenues. Late EB-MB tombs have also yielded some metal weapons (axe, spear point and bronze dagger), and semi-precious stone (carnelian, agate, etc.) artefacts from both distant and more nearby sources. The deposition of prestige objects seems to characterize increasing social complexity−sometimes defined as a class of ‘warriors’−at that time, and the development of a shared social code in the Near East and Arabia, in a context of increased mobility ([58–60]). This phenomenon probably correlates with changes in the morphology of the tombs in other nearby regions−the apparition of cruciform tombs and rectangular collective tombs (e.g., [27, 61, 62])−indicating profound societal modifications in western Arabia, possibly again in link with sedentarization processes in regional oases.

#### Function, local economy and long-distance trade

The observations on settlement type and density at the al-Natah site necessarily raise questions about subsistence and the function of the site in the Hijaz during the Bronze Age. First, there can be little doubt that the purpose of the walled oasis of Khaybar must be intricately linked to its significant natural water capacities (springs and aquifers), agricultural production, and husbandry activities, which brought stability and a steady and secure food supply to its inhabitants. The outer fortifications were thus clearly designed to protect the site’s resources and to regulate access and trade. Although bioarchaeological studies are still in an early stage, it is likely that the vast expanses of well-watered land within the walled oasis permitted the production of significant yields and ensured food stability for sedentary populations, as shown by the presence of cereals at that time (Bouchaud and Chambraud pers. comm.; [35]: Table 1). This paleoenvironmental context is moreover further supported by Bronze Age data from Tayma [63]. The impressive quantity of animal bones in the dump in SD3 suggests a high consumption of meat and milk (ovi-caprines in particular, Monchot pers. comm.), and therefore that animal husbandry was part of pastoral activities in and around the oasis.

**Fig 15:**
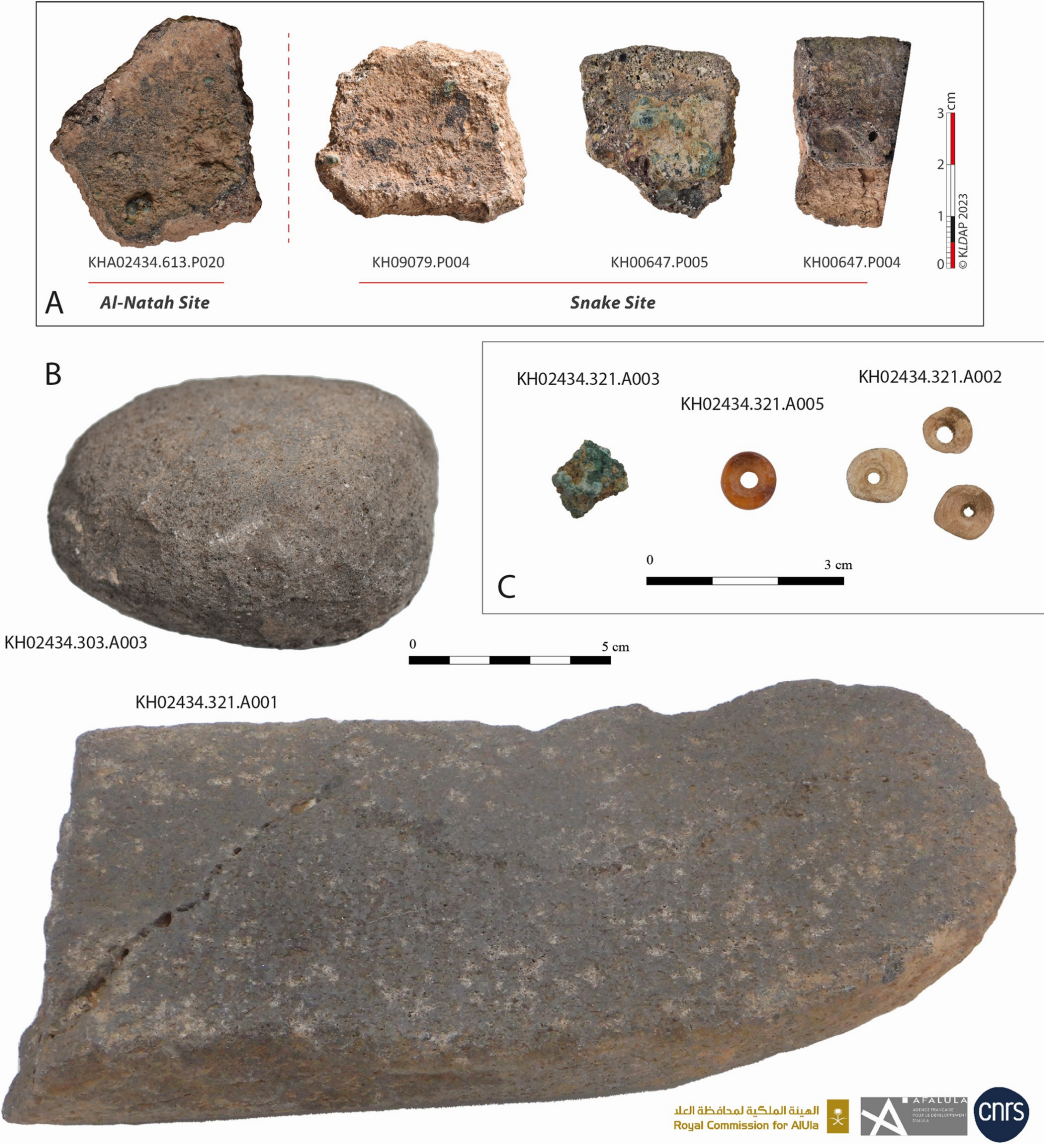
Types of archaeological objects found at al-Natah (KH02434) and at Snake site (KH09079, KH00647: A. fragments of ceramic crucibles with metallic remnants on the inside, B. pestle and mortar, C. bone and carnelian beads and fragment of metal. Reprinted under a CC BY license, with permission from AFALULA-RCU-CNRS, 2024.

**Fig 16:**
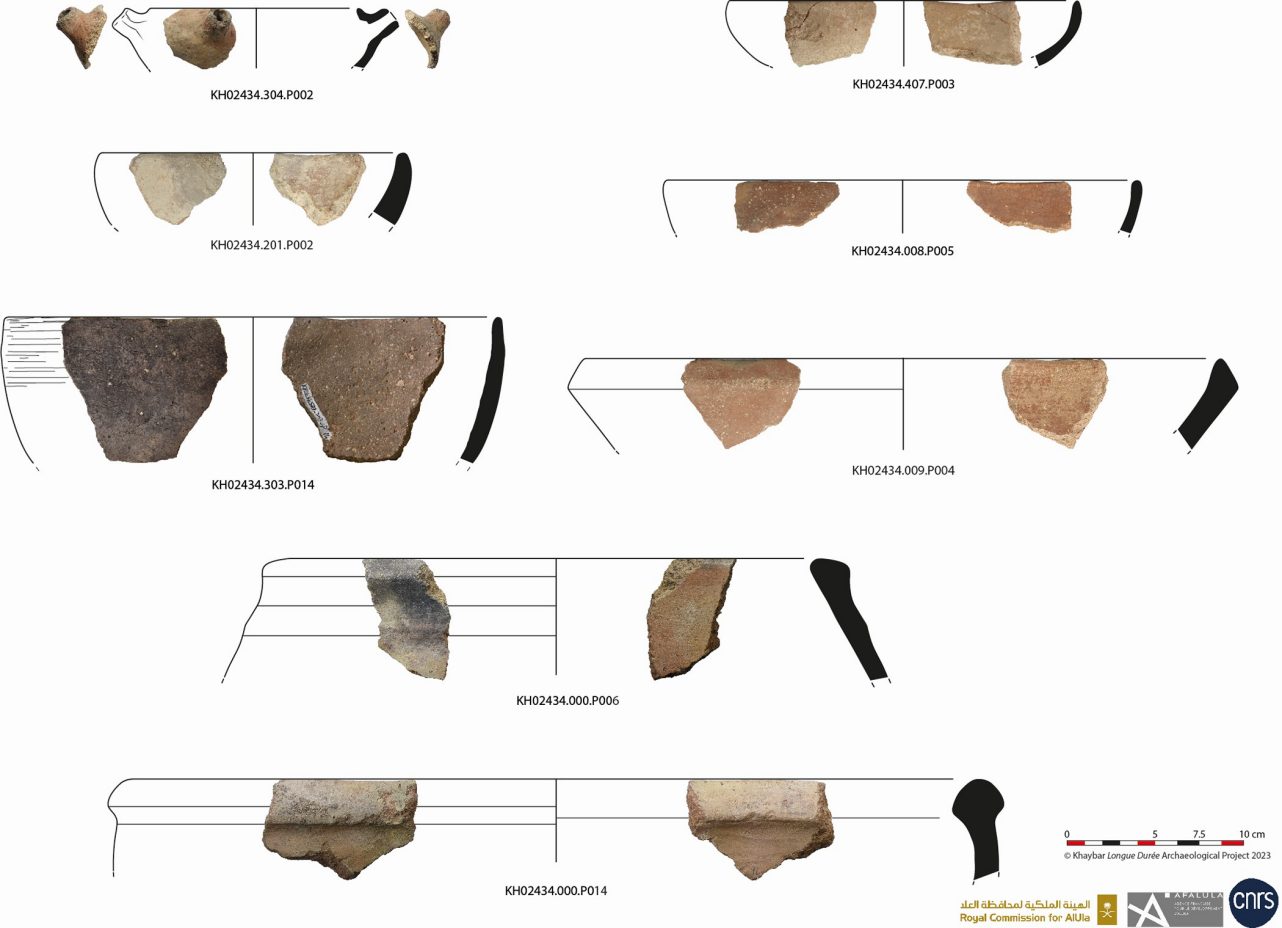
Late Early Bronze Age-Middle Bronze Age vessels from the al-Natah site.

The excavations also revealed the presence of a large quantity of small and medium-sized basalt grinding stones, mortars and pestles on the surface of the site and in the dump layer of SD3 (Fig 15). These locally made objects were probably used to grind cereals ([35]) and prepare meals in domestic contexts. Pottery tableware consisted mainly of simple open forms, bowls and jars, sometimes with inset rims, rarely decorated, but generally covered with burnished red slips (Fig 16). Bowls were also primary cooking vessels. Closed vessels, such as jars and pithoi, indicating the storage of foodstuffs were also found on site but are rare. These finds, of very simple shapes found all over the site and in the oasis, suggest a relatively egalitarian society, with local workshops dedicated to craft activities.

Alongside this local economy, the site was part of a wider regional exchange network, at a time when trans-Arabian travel by donkey was on the increase ([29, 61]). The microfabrics of a few rare sherds of Red Burnished Ware found during surveys and excavations seems to come from outside the oasis (perhaps Qurayyah or Tayma). Sourcing analyses of arsenic copper at Tayma and Qurayyah have shown a regional provenance, either from Oman or the Arabian Shield ([60]: 141, [64]). On the Qurayyah plateau, a metallurgical workshop set up in a large reused older tower dates back to the 22^nd^-20^th^ centuries BCE, before intense activity at the end of the Middle Bronze Age and the beginning of the Late Bronze Age ([60]). We would, therefore, be inclined to assume that well-known copper mines in the Hijaz were already in use during the late EB-MB. Four pottery sherds of crucibles with metal remains preserved on their internal walls were found in Khaybar Oasis (al-Natah and the Snake sites), support the hypothesis of local metallurgical production and a possible but relative intensification of trade. This still very modest evidence confirms that copper alloys were processed in the oasis and that they may have been part of a long-distance trade network.

On a regional scale, metallurgical production and food surpluses could account for the construction of several walled oases around Khaybar (Hait, Huwayyit, al-Ayn and al-Tibq oases, in particular Fig 1). Our recent visits in these oases confirm similar rampart models with adjoining bastions and the presence of very comparable Red Burnished Ware. The existence of this medium-distance network, centered on the Harrat Khaybar (Harrat al-Nar) around 2400–1500 BCE, and already connected by funerary avenues ([44]), should be more closely considered in the future. We cannot rule out the possibility that Khaybar‒and the site of al-Natah‒was a local political and economic center, perhaps in a functional ‘hierarchical’ relationship, as part of a core-periphery interaction?

## Discussion

Can al-Natah, and more widely, Khaybar walled oasis, be considered as a Bronze Age ‘urbanized’ site? This question needs to be addressed cautiously, given the risk of under- or over-evaluating a long-term indigenous socio-economic development.

### Walled oases and the issue of early urbanization in Northwestern Arabia

In the case of Bronze Age walled oases and urbanization in Northwestern Arabia, it should be remembered that in-depth investigation of the region is only just beginning and that knowledge of residential contexts of pre-caravan kingdoms, remains minimalist, due to noticeable biases in archaeological research. Thus, a debate on the rise of social complexity and urbanization in this context can only be preliminary.

Recently, Charloux, al-Malki and al-Qaeed proposed considering the emergence of a north Arabian walled oases ‘phenomenon’ at Tayma and Qurayyah as an Early Bronze Age phenomenon peripheral to the first urbanization of the Southern Levant ([33]: 282–283). In addition to the contemporaneity of the fortification process this proposal was based on a body of concordant evidence of long-distance contacts in the first half of the third millennium BCE: increased pottery production, intensive agriculture of Mediterranean tree crops (olive, grape, etc.), long-distance contacts through the use of donkeys, the development of a local power relying on a labor force for monumental architectural activity that could well evoke the largescale collective works of prosperous early southern Levantine urban centers.

In the same year, Luciani described Qurayyah, Tayma and Khuraybah/AlUla as ‘urban’ oases’ [4]: "very extended permanent settlements in the desert, featuring monumental architecture, complex irrigation and agricultural schemes, pottery and metallurgical production" [4]. These criteria would define a form of urbanization based on local criteria and a ’functional’ perspective, thus attempting to avoid the risk of confusion with Levantine and Mesopotamian urban concepts. For these two proposals, the main difficulty lies in the lack of archaeological data and of a precise chronology, and particularly on the absence of excavated residential areas.

Recent discoveries at Khaybar now provide a first image of a settlement associated with ramparts from this period and allows better assessment of the two preliminary proposals. The al-Natah site is a small settlement of 2.5 ha with an enclosed surface area of only 1.6 ha protected by a wide rampart to the south−perhaps during a second phase−with a population of around 500 inhabitants. The internal organization of the site is subdivided by a rampart into two distinct functional zones: residential and ‘central’ (perhaps decision-making) area. There is an absence of overall planning, but there is a connected or isolated network of 2-m-wide streets between buildings built according to a recurring architectural model that can be adapted to topography and needs. Evidence of local production−pottery and metalwork−testifies to craft specialization exceeding the domestic sphere. Although the finds are mainly commonplace, a number of prestige artifacts and deep changes in funerary practices bear witness to long- and medium-distance contacts and increased social differentiation. Monumental outer and inner ramparts bear witness to collective works organized by a central authority around a central strategic and decision-making core dominating the cultivated area and the main circulation routes in the oasis. The stages of the ramparts and funerary developments in Khaybar testify to an increased need for protection, control and ostentation, and are probably linked to social stratification at the site over time.

**Fig 17:**
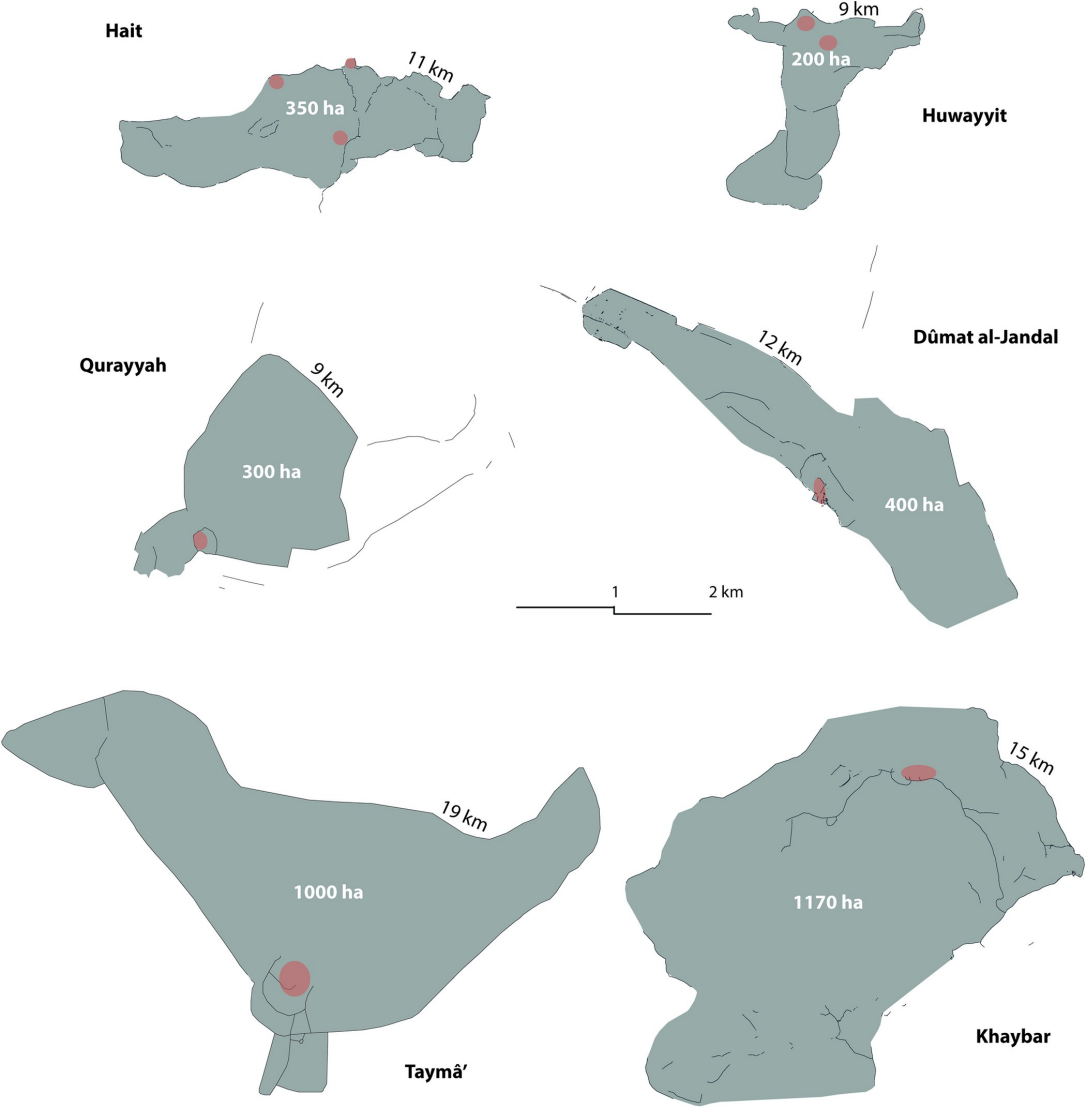
Comparison of the dimensions of the main Bronze Age walled oases−and their settlements in red−in Northwestern Arabia (except iron age Dumat al-Jandal).

Archaeological data for other large walled oases from Bronze Age appear to be broadly comparable to those from Khaybar, in terms of oasis organization, collective architectural investment, diet, technology and socio-economics ([63, 65]). However, it remains difficult to describe accurately the emergence of the fortified agglomerations of Qurayyah and Tayma. Monumental outer walls (19 km long at Tayma and ca. 9–13 km at Qurayyah; Fig 17) are dated to a large time-span around the first half of the third millennium BCE with later modifications ([16]), through OSL, ^14^C and pottery dating [4, 34], but with limited chronological precision. The existence of an installed hydraulic networks in the walled oasis of Tayma [66] and at Qurayyah ([30]) confirms vast areas of organized and sustained cultivation. In the third and early second millennia BCE (Phases T10 to T9), the Tayma oasis enjoyed a mixed agro-pastoral economy based on a sheep/goat diet that also included cattle and equids ([65]: Tab 3) and on cereal (barley, etc.) and horticultural resources (vines, figs, etc.) from a vast terroir ([63]). Excavations of tombs with funerary goods also point to the creation of local elites at the beginning of the second millennium BCE ([26, 60, 67]), while local pottery and metallurgical production confirms that craftsmanship levels and regional interconnectivity were fairly comparable to those of Khaybar ([4, 68]). Unfortunately, our knowledge of the residential contexts is practically non-existent for Qurayyah ([4, 60]) and limited to only several architectural remains for Tayma ([16], [69]) and AlUla (Dedan: J. Rohmer, pers. comm.). At Tayma, however, the excavation of a 65 sq. m. quadrangular building (E-b5) with a central corridor serving 10 small rooms, and another vast multi-period building to the south (E-b15), testifies to the existence of large constructions with a collective function in the Early Bronze Age and redeveloped in the Middle Bronze Age ([66, 69, 70]: 113, 116; [26]: 137, 153‒154).

At the present stage, we suppose that the residential areas in Tayma and Qurayyah must have been roughly in the same size range as al-Natah. Although not yet reached, the EB-MB residential area of Qurayyah could probably not have exceeded the size of the fortified central zone (6 ha), and especially the 3 ha of the central tell within this zone. The Tayma residential area could potentially have been larger, but this remains uncertain in the absence of finds in sectors P, D, F and Z. It could therefore have been limited to the center of the site (sectors E and W) and have been surrounded by uninhabited areas, perhaps in order to reserve space for future construction or to accommodate endangered neighboring populations and their herds from other mobile groups (e.g. in the Southern Levant: [71]: 86, 87 n4). Considering their sizes, al-Natah, and most probably the Tayma and Qurayyah settlements would be considered as villages in contemporary Mesopotamia or Egypt, whereas researchers working in the EB-MB Southern Levant would place them in the category of medium-sized settlements (category C: between "1.1 and 4.9 hectares mean 3 hectares" ([55], [71]see also [72]: 22). By comparison, some researchers claim that a fortified site of 1 hectare (therefore smaller than al-Natah) should be considered ‘urban’ as early as the Early Bronze I period in the Southern Levant (e.g. [73]: 253).

### The Bronze Age Southern Levant benchmark

The long debate on urbanization in the Southern Levant provides interesting benchmarks for archaeological and theoretical comparisons with the situation in Northwestern Arabia, not only because of their geographical proximity but also because of the development of comparable subsistence modes in the desert margins and of their political and social organizations. Moreover, contacts and interactions with EB Southern Levant are supported by the recent discovery of Southern Levantine style pottery and lithics in al-Bad‘ [74, 75] and Qurayyah [62].

Over the past forty years, researchers working in the Southern Levant have developed a range of theories and models−chiefdoms, kingdoms, and city-states, ‘secondary states’, ‘corporate villages’, ‘townism’, ‘slow urbanism’, etc.−in an attempt to explain and characterize the indigenous socio-economic complexity from the fourth to the third millennium BCE ([35, 38]). In this discussion, correlated with the concept of urbanization, the Syro-Mesopotamian urban model has long been the main comparison (on this issue see [38]). Often characterized by the rise of city-states, it is generally defined by large interconnected settlements with a concentrated population monitoring a wide rural landscape, comprising complex central institutions, strong social stratification controlling economic production and surplus, craft and agricultural specialization, and the use of writing and administrative tools ([6]). On this theoretical basis, two main antagonistic approaches−pro- and anti-urban/ized−with many nuances and divergences, have competed in the Southern Levant for over forty years:

On the one hand, researchers working in the western part of the Southern Levant have often favored a ‘pro-urban’ or ‘urbanized’ position. Although regionalized and of a modest scale, this indigenous urbanization−more or less inspired by external Egyptian and Syro-Mesopotamian spheres, depending on the scholar−is frequently considered to be a local and low magnitude variation of Syro-Mesopotamian urbanization, with its own singularity. The first southern Levantine ‘urbanization’ occurring as early as the EB I ([76–78]) or at the very beginning of the EB II ([7, 79, 80], is thought to have been characterized from its first phase by fortified towns and the presence of out-sized buildings within ‘modest aggregations of people living within nucleated settlements organized into complex hierarchical societies’ ([78]: 267). The discovery of large public buildings, such as the granary at Beth Yerah or the palace at Yarmouth in the first half of the third millennium BCE (EB II & III, around 3100–2500 BCE [81]), and the presence of seals, would provide evidence of the management of the means of production by an elite, and the rise of city-states in pre-literate southern Levantine society.

On the other hand, several scholars working in the eastern part of the region, and particularly in the desert margins, have often tried to adopt a theoretical approach focusing on non-evolutionary positions, generally favoring the variety of heterarchical organizations of indigenous societies, which were nevertheless capable of large-scale collective work ([37, 38, 82]). Philip does not only oppose the differences in scale and nature of the models of the complex based polities of Western Asia with the situation of the fortified sites in the Southern Levant ([38]). As a whole, he also rejects a perspective focused on regional centers dominated by an elite in favor of more complex and varied forms of organization at different scales, which are also capable of exercising power. In parallel, Savage, Falconer and Harrison strongly refute the city-state hypothesis for the EB Southern Levant ([82]).

Probably one of the most interesting examples of the ambivalence of viewpoints is reflected by the interpretation of the site of Jawa in the hyper-arid desert of eastern Jordan. This site, which shares multiple traits with Bronze Age walled oases in Northwestern Arabia, has long focused the ambiguity of defining urbanization in the desert margin of the Southern Levant. Measuring 10 ha, the Jawa fortified settlement from the second half of the fourth millennium BCE features elaborate hydraulic systems, the result of advanced water catchment and management technology ([83–85]: 91; [86, 87]), a double rampart with gates and towers, and a relatively dense settlement (population estimated at around 1,500 individuals) connected by alleyways, but with no trace of visible social differentiation in the buildings or archaeological material. According to Braun, these arguments “are indubitable evidence of urbanism, especially in light of the degree of planning and logistics necessary to create a settlement of such proportions within an arid zone” [78]. Helms described the site as a ‘desert city’ ([85]). This identification is challenged by Nicolle and Braemer, who consider that Jawa−like other large fortified settlements of the EB I period (c. 3700–3100 BCE, according to [81])−is a ‘pre-urban’ gathering site for mobile pastoralists at the heart of exchange and distribution networks in the heart of the desert [80]. For these latter authors, the ‘urban’ stage would not have been reached until EB II (3100–2900 BCE) in the Syro-Jordanian region, with a clear qualitative change in social organization, as at Labwe and Khirbet al-Batrawy, for example. Precisely on the same criteria of specialization, work distribution and management, Müller-Neuhoff ([88]) describes Jawa, and the other hillforts in the region, with a non-egalitarian and heterarchical organization model, according to the approach developed by Chesson and Phillip ([37]).

These opposing scientific positions on a same site highlight on the difficulty of describing and characterizing indigenous socio-political process and emergence of urbanism in peripheral core regions and particularly in desert margins area during the early Bronze Age. The position of the cursor depends, above all, on the extent to which and how the socio-political integration of the site, elite and regional networking are highlighted. Recently, Chesson ([36]) has proposed an interestingly ‘middle way’, defined as ‘townism’ or ‘slow urbanism’, to reconciliate the two antagonist approaches for multiscalar contexts, characterized by: “1. Localized population aggregation and scalar variability; 2. Intensification of agropastoral staple food production to feed larger communities; 3. Increasing but limited specialization of craft production and regionalism of certain crafts; 4. Reworking notions of kinship and corporate groups, with little evidence for significant social differentiation in residential contexts” ([36]: 179, Table 9.2). This ‘middle way’, distinct from ‘real’ urbanization [36], could be an interesting way of describing the Bronze Age walled oases in Northwestern Arabia.

These theories and concepts on the Early Bronze Age urbanization in the Southern Levant, take shape in particular contrast to the non-urban phase that follows, the Early Bronze Age IV (2500–2000 BCE). This period is often considered as a ‘collapse’ or ‘resilience’ stage, after the urbanized EB II-III ([7, 89]). This ‘post-urban’ period is generally characterized by the presence of villages and smaller rural centers. It was often believed that EB IV social organization, strongly impacted by aridification of the climate, showed weaker social integration on a regional scale. However, there is nowadays growing evidence of cultural and sedentary continuity in several sites, notably in Transjordan ([8, 39, 89]). Many ‘urban traits’ continue (fortifications, temples, evidences of hierarchies, etc.) [39, 90], and tend to blur the distinction of socio-political complexity with the previous urbanized period. Today, this inevitably brings into questions the pertinence of such singular semantic designations, without taking into account microregional variations and grasping “the nature, scale and diversity of the society” [39]. Richard proposes the use of the concept of ‘rural complexity’ for defining “low urbanization, with few centers and agrarianism with far-away cities” during the EB IV.

The emergence of a new phase of urbanism during the first half of the second millennium BCE (2000–1600 BCE, MB) puts an end to this long theoretical debate on the nature of early local urbanization. This ‘second’ urbanization is characterized by fortified tells, city walls, fortresses, palatial and cultic structures, temples, etc. The advent of written documents and the intensity of trade with the Syro-Mesopotamian and Egyptian worlds, signal a “peak of independent Canaanite cultural, political and social development” ([7]: 180–185), quite different from the previous periods.

These benchmarks on early urbanism in the Southern Levant show the necessity of contextualizing the variations of socio-political complexity according to time, geography and environmental evolution.

### Defining a process of ‘low urbanization’ in the Northwestern Arabian desert

In the first half of the third millennium BCE, it is conceivable that the walled oases of Tayma and Qurayyah were inspired by the ‘first urbanization’ in the Southern Levant, with which they certainly had contact [33, 62]. The construction of large enclosures surrounding vast rural areas, and the erection of monumental buildings in probable residential areas ([16, 69]), as well as the presence of Mediterranean plants, suggest an adaptation of the southern Levantine situation, but according to distinct environmental and cultural criteria. The lack of data does not presently allow us, however, to specify the degree of socio-economic integration of these sites.

From the second half of the third millennium BCE, groups of mobile pastoralists that constructed funerary avenues, sedentarized and erected small towns in oases in the Harrat Khaybar region. The nucleated settlement of ‘tower’ type houses with wide basalt basements, evoke a local Arabian tradition of dwelling’s construction. Around 2250–1950 BCE, the oasis was gradually protected by a first outer rampart, then a bit later in the early second millennium BCE by a second fortification curtain−notably for the town of al-Natah. Through this collective labor, the authority strengthened group cohesion and participated in a communal identity. It clearly placed an emphasis on ostentation and control of resources, probably at a time of increasing social stress due to climatic changes (4.2. BP event, [47–49]). The evolving socio-political situation at the turn of the second millennium is indeed confirmed by a change in funerary practices, the appearance of stepped tower-tombs containing prestige goods, the existence of inter-site functional subdivision−and the erection of collective buildings at Tayma [16, 69]. Agricultural technology and local craft production, different from Southern Levant production, developed at that time, in parallel with the continuation of pastoral activities. At the regional level a complex of multiple walled oases in Harrat Khaybar (Khaybar, Hait, Huwayyit, al-Ayn and al-Tibq oases) also probably emerged, connected by funerary avenues that were still in use, in parallel to the occupied walled oases of Qurayyah and Tayma. This evidence suggests the progressive rise of a central authority in the walled oases, better social integration through time, the emergence of a dominating class and increasing long-distance exchanges, as well as local traditions of construction and craft production. This autochthonous transitionary context, that we here describe as ‘low urbanization’, contrasts sharply with the contemporaneous MB Levantine and Mesopotamian urban situations.

## Conclusion

The new archaeological evidence from Khaybar introduced in this article confirms a stage of major socio-economic transition from a mobile way of life to sedentarization and town life between the second half of the third millennium and the early second millennium BCE (prior to abandonment of Khaybar in the mid second millennium BCE). This radical change in life style, from a pastoral-nomadic way of life to more agro-pastoral subsistence, and in mortuary practices (from funerary avenues to site necropolis), had a profound impact on autochthonous socio-economic organization and complexity.

Considering previously developed arguments, a weak hierarchical political organization could well characterize these Northwestern Arabian walled oases during the Early and Middle Bronze Ages. In a context of small settlements with limited population, controlling large agricultural landscapes but lacking writing and administrative tools, the ‘low urbanization’ southern Levantine concepts (‘slow urbanism’ or ‘rural complexity’, etc.) help to describe the transitional indigenous political and socio-economic situation.

Although more excavations in Khaybar and other Bronze Age settlements in the region are needed to assess the level of socio-economic complexity during this period in more detail, we can affirm rather confidently that, alongside non-sedentary groups in the desert, the settlement morphology in Northwestern Arabia during the third millennium and early second millennium BCE, was characterized by the existence of small towns, such as the al-Natah site, within huge interconnected walled oases.
